# Personalized bioceramic grafts for craniomaxillofacial bone regeneration

**DOI:** 10.1038/s41368-024-00327-7

**Published:** 2024-10-31

**Authors:** Ana Beatriz G. de Carvalho, Maedeh Rahimnejad, Rodrigo L. M. S. Oliveira, Prabaha Sikder, Guilherme S. F. A. Saavedra, Sarit B. Bhaduri, Debby Gawlitta, Jos Malda, Darnell Kaigler, Eliandra S. Trichês, Marco C. Bottino

**Affiliations:** 1 Department of Cariology, Restorative Sciences and Endodontics, School of Dentistry, University of Michigan, Ann Arbor, MI USA; 2 Department of Dental Materials and Prosthodontics, São Paulo State University, São José dos Campos, SP Brazil; 3 Federal University of São Paulo, Institute of Science and Technology, São José dos Campos, SP Brazil; 4 Department of Mechanical Engineering, Cleveland State University, Cleveland, OH USA; 5 Department of Mechanical, Industrial and Manufacturing Engineering, University of Toledo, Toledo, OH USA; 6 Department of Oral and Maxillofacial Surgery & Special Dental Care, University Medical Center Utrecht, Utrecht University, Utrecht, The Netherlands; 7 Regenerative Medicine Center Utrecht, Utrecht, The Netherlands; 8 Department of Clinical Sciences, Faculty of Veterinary Medicine, Utrecht University, Utrecht, The Netherlands; 9 Department of Orthopedics, University Medical Center Utrecht, Utrecht, The Netherlands; 10 Department of Periodontics and Oral Medicine, School of Dentistry, University of Michigan, Ann Arbor, MI USA; 11 Department of Biomedical Engineering, College of Engineering, University of Michigan, Ann Arbor, MI USA

**Keywords:** Biotechnology, Translational research

## Abstract

The reconstruction of craniomaxillofacial bone defects remains clinically challenging. To date, autogenous grafts are considered the gold standard but present critical drawbacks. These shortcomings have driven recent research on craniomaxillofacial bone reconstruction to focus on synthetic grafts with distinct materials and fabrication techniques. Among the various fabrication methods, additive manufacturing (AM) has shown significant clinical potential. AM technologies build three-dimensional (3D) objects with personalized geometry customizable from a computer-aided design. These layer-by-layer 3D biomaterial structures can support bone formation by guiding cell migration/proliferation, osteogenesis, and angiogenesis. Additionally, these structures can be engineered to degrade concomitantly with the new bone tissue formation, making them ideal as synthetic grafts. This review delves into the key advances of bioceramic grafts/scaffolds obtained by 3D printing for personalized craniomaxillofacial bone reconstruction. In this regard, clinically relevant topics such as ceramic-based biomaterials, graft/scaffold characteristics (macro/micro-features), material extrusion-based 3D printing, and the step-by-step workflow to engineer personalized bioceramic grafts are discussed. Importantly, in vitro models are highlighted in conjunction with a thorough examination of the signaling pathways reported when investigating these bioceramics and their effect on cellular response/behavior. Lastly, we summarize the clinical potential and translation opportunities of personalized bioceramics for craniomaxillofacial bone regeneration.

## Introduction

Craniomaxillofacial bone defects arise from various etiologies such as traumas, fractures, surgical interventions, tumor resections, infections, or congenital malformations.^1–5^ An established approach for addressing substantial bone defects and facilitating tissue reconstruction involves the utilization of bone grafts.^1,6–9^ Fundamentally, these graft materials function as scaffolds to support the formation of new bone, ensuring the preservation of appropriate spatial conditions and mitigating the infiltration of undesirable tissues into the affected region.^10^

Bone regeneration constitutes a complex phenomenon encompassing molecular, biochemical, and mechanical dimensions.^11^ From a clinical perspective, distinctions between maxillary and mandibular bones arise from the varying mechanical stresses and muscle tensions to which these bones are subjected, potentially yielding divergent behaviors.^12^ Consequently, the unique attributes of mandibular bone, including its distinct origin, function, and composition, present challenges in reconstruction and replacement.^6^ Nonetheless, autografts have been widely acknowledged as the gold standard.^1^ Fibula flap autografts have been an option for reconstructing maxillofacial defects.^6,13,14^ While autografts offer significant advantages, such as optimal osteogenic, osteoconductive, and osteoinductive potentials,^1^ they also present drawbacks, including limited availability and quantity and the necessity of two surgical sites, leading to increased pain and infection rates.^1,6,15^ In light of the challenges linked to current graft options, additive manufacturing (AM) is explored to fabricate bone-like substitutes for craniomaxillofacial bone applications.^16^

AM technologies, including vat photopolymerization, material extrusion, material jetting, and direct energy deposition, have emerged as promising alternatives for bone tissue engineering.^1,17^ These technologies enable the production of personalized grafts utilizing a diverse array of biomaterials and allow the fabrication of porous and intricate three-dimensional (3D) geometries with a high level of precision that closely mimics the architecture of bone.^6,13,18,19^ Extrusion-driven techniques are the preeminent printing method,^20^ wherein a continuous strand of the specified biomaterial is extruded onto the collecting platform based on a computer-aided design.^18,21^ In this review paper, a particular emphasis is placed on extrusion 3D printing of bioceramics since these materials are notably recognized for their capability to produce grafts with mechanical properties, structure, and composition similar to native bone, thereby augmenting osteogenic competency.^11^

This review represents a major effort in the field of regenerative dental medicine by covering a broad spectrum of topics related to the development of bioceramics and the use of 3D printing technologies to generate personalized bone grafts. To the best of our knowledge, the present review provides, for the first time, a comprehensive appraisal of the literature that spans all classes of bioceramics, explores advanced 3D printing techniques used in the fabrication of bioceramic grafts, and examines the dynamic interactions between cells and these materials. Furthermore, this paper offers an in-depth exploration of how 3D printing can revolutionize regenerative dental medicine, particularly emphasizing the development stages and clinical implementation of personalized bioceramic craniomaxillofacial grafts. It meticulously addresses factors affecting the printing process, such as material selection and the direct ink writing (DIW) technique. It also covers scaffold design in detail, considering macro- and microstructural aspects and their impacts on biological responses.

Significant attention is devoted to the feasibility and benefits of creating personalized grafts, showcasing how this innovative approach could revolutionize craniomaxillofacial bone reconstruction. While the precision of AM in producing tailored bone grafts is a significant advantage, the path from development to clinical application remains an area of active exploration. To that end, this review highlights well-established in vitro models and provides a thorough examination of the signaling pathways relevant to bioceramics and their influence on cellular function and bone formation. It concludes by summarizing the potential of next-generation personalized bioceramics in craniomaxillofacial bone regeneration.

## Bioceramics for bone regeneration

**Fig. 1:**
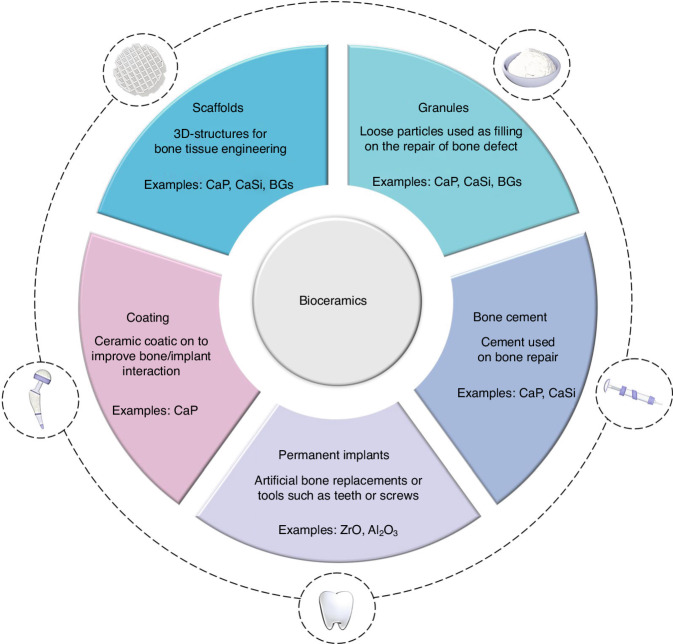
Schematic of different types and applications for bioceramic materials

Bioceramics are a crucial class of materials with numerous applications, including repairing and reconstructing damaged bone.^22,23^ Based on their tissue response, bioceramics can be classified into three prominent categories: nearly inert (e.g., alumina and zirconia), bioactive (e.g., bioactive glass), and bioresorbable ceramics [e.g., β- and α-tricalcium phosphate (TCP)].^22,23^ Various biomedical products can be manufactured using bioceramics in their different forms, such as powders for controlled-release drug delivery systems, granules for bone grafting, solid pieces for dental implants, and porous structures (scaffolds) for tissue engineering applications^24,25^ (Fig. 1). In clinical settings, various products for bone substitution are frequently utilized, including granules, blocks, and injectable putty. Table 1 provides a summary of these products, categorized by the main bioceramic classes and their respective applications. However, these products are prefabricated, and with the recent development of technologies, there are new possibilities for personalized bone grafts. This topic reviews the main bioceramics reported for bone repair and regeneration research.

**Table 1:** Different classes of bioceramic materials available in the market and their clinical applications^a^

Classes	Product name	Company	Composition	Clinical application
Calcium phosphates	MasterGraft®	Medtronic	β-TCP + HA	Spine and Orthopedic grafts
	Dental adbone®TCP	Medbone®	β-TCP	Craniofacial and orthopedics grafts
	BoneSource®	Stryker orthopedics	Tetracalcium phosphate and dicalcium phosphate anhydrous	Cement for Orthopedic
Bioactive glasses	Bonalive®granules	BonAlive	S53P4	Bone cavity filling, osteomyelitis, and mastoid cavity
	NovaBone Porous Granules	NovaBone®	45S5	Craniofacial grafts
	Glassbone® Granules	Noraker®	45S5	Orthopedic and craniofacial grafts
Calcium silicate	MTA Fillapex	Angelus Solucoes Odontologicas	Mineral trioxide aggregate, salicylate resin, natural resin, bismuth, and silica	Bone cement sealer
	ProRoot	Dentsply Sirona	Mineral-trioxide-aggregate	Craniofacial grafts
	BIO-C® TEMP	Angelus Solucoes Odontologicas	Tricalcium Silicate, Dicalcium Silicate, Tricalcium Aluminate, Calcium Oxide, Calcium Tungstate-based Resin, Polyethylene Glycol, Titanium Oxide	Bone cement sealer
Composites	Bonalive®putty	BonAlive	S53P4/ PEG	Filling of bone voids and gaps
	OssiMend® Bioactive	OssiMend®	Carbonate apatite anorganic bovine bone mineral, 45S5 bioactive glass, and Type I Collagen.	Spine and Orthopedic grafts
	Bi-Ostetic™ Bioactive Glass Foam	Berkeley Advanced Biomaterials	Type I bovine collagen, 45S5 bioactive glass, HA and TCP	Orthopedic grafts

^a^Information obtained according to the respective manufacturers

### Calcium phosphates (CaPs)

Calcium phosphates (CaPs*)* are the most widely used bioceramics for craniomaxillofacial bone regeneration. Due to their chemical composition, similar to bone’s mineral phase, they show high biocompatibility and excellent osteoconductivity and osteoinductivity (in certain conditions).^24,25^ They can be classified according to the calcium-to-phosphorous ratio (Ca/P atomic ratio).

Synthetic hydroxyapatite (HA, Ca_10_(PO_4_)_6_(OH)_2_) (Ca:P = 1.67) has excellent osteoconductivity and osseointegration properties.^26^ However, due to its low mechanical properties and low biodegradability, HA is used more frequently as a bone filler (cement or granules) and coating on metallic prostheses instead as a scaffold.^6,24,27^ HA can be combined with natural or synthetic polymers to enhance the polymers’ cell adhesion properties and bioactivity. On the other hand, polymeric materials provide excellent flexibility and biodegradability to the scaffold, and in this new configuration, they can be used for regenerative applications.^26^

Tricalcium phosphates (Ca_3_(PO_4_)_2_, (Ca:P = 1.5)) have four polymorphic forms: β (rhombohedral), α (monoclinic, and occurs above 1 115–1 150 °C), α‘ is a high-temperature phase (occurs above 1 430–1 470 °C), and γ a high-pressure phase.^28–30^ The α- and β- phases are the most commonly used. The α-phase is more soluble in aqueous systems than β-TCP and is used to prepare calcium phosphate cement.^31,32^ The β is interested in bone defect treatment due to its high resorption rate compared to HA and thermal stability at room temperature. β-TCP powder is also easily attainable by different routes (solid-state reaction and thermal conversion) and shows high biocompatibility.^30,33^

Dicalcium phosphate anhydrous (DCPA, CaHPO_4_), or monetite, is another CaP widely applied to bone regeneration. It can be obtained through the dehydration of dicalcium phosphate dihydrate (named brushite, DCPD, CaHPO_4_·2H_2_O) using solvent-based methods or external energy-assisted processing.^34^ Its main application is as bone cement; however, it can be applied as a coating on metallic implants, granules, injectable pastes, and scaffolds.^34^

HA has been on the market since the 1980 s, including applications in dental surgery, spine surgery, maxillofacial repair, and more.^35,36^ Thus, there are many examples of CaPs available for clinical procedures. The most applied are β-TCP, HA, and biphasic calcium phosphate (BCP), a combination of both. Commercially available HA (Regenos^®^) and β-TCP (Affinos^®^) scaffolds with unidirectional pore structures are suitable grafts for trauma, spine, and benign tumor removal surgeries.^37^ 3D-printed HA has shown potential as a bone graft for alveolar ridge preservation in a randomized clinical trial, equivalent to commercially available bone graft (NanoBone^®^).^38^ Granules and paste of BCP are suitable for human maxillary sinus bone augmentation in a randomized clinical trial, leading to the regeneration and maturation of new bone after six months of bone healing.^39^

### Calcium phosphate cement (CPC)

Calcium phosphate cements (CPCs) have been extensively applied in bone tissue repair/regeneration due to their good biocompatibility, biodegradability, and excellent stimulation of osteogenesis. They are self-setting materials obtained through reactions between a CaP powder and a hydraulic reactive solution. After being mixed into a paste, they self-induce dissolution-precipitation reactions that are responsible for its hardening. The end product formed after the cement precipitation will depend on the starting powder and the solution employed in its production. There are three different categories of CPCs, according to their end products: apatite cements (CDHA - calcium-deficient hydroxyapatite, Ca_10_ − x(HPO_4_)x(PO_4_)_6_ − x(OH)_2_ − x, 0 < x < 1 or HA), brushite cements (DCPD, CaHPO_4_·2H_2_O), and monetite cements (DCPA, CaHPO_4_).^40,41^ CDHA can be obtained, for instance, through the reactions of α-TCP and water or phosphate buffer.^42,43^ DCPD can be obtained by combining β-TCP and phosphoric acid.^43^ DCPA is the anhydrous form of DCPD and can be obtained through dehydration or by adjusting brushite cement reactions to a deficient pH condition. ^34^ Taken together, in the last two decades, many formulations of CPCs and combinations with different materials were developed to produce 3D-printed scaffolds with controlled shapes and porosity that can be used directly in craniomaxillofacial bone reconstruction.^40,44,45^

CPCs began to be developed in the 80s,^46^ being already widespread in dental and orthopedic applications. Several powder mixtures are commercialized for clinical applications, including DCPD, α-TCP, and β-TCP amorphous calcium phosphate (ACP).^47^ Nowadays, CPC research focuses on producing 3D-printed grafts/scaffolds, using CPCs as drug carriers,^48^ and producing macroporous injectable cement.^49^

### Bioactive glasses (BGs)

In the late 1960s, bioactive glass (45S5 Bioglass^®^, 45SiO_2_–24.5Na_2_O–24.5CaO–6P_2_O_5_, wt%) was developed by Hench and collaborators.^23^ This material can rapidly form strong chemical bonds with soft and hard tissues, and it is attributed to the hydroxycarbonate apatite (HCA) formation on its surface, following the ionic dissolution (e.g., Si, Na, Ca, phosphate ions, etc.).^22,50^ Nowadays, there are several compositions of bioactive glass: silicate-, phosphate-, and borate-based glasses,^51,52^ and they can find many biomedical applications. Traditional melting-quenching or sol-gel techniques can be used to produce them.

Mesoporous bioactive glasses (MBGs) are another class used for bone regeneration due to their excellent bioactive properties (formation of a surface HA layer within a few hours from contact with biological fluids).^24,53^ They have a porous structure with pores ranging from 2 to 50 nm, classified as mesoporous by the International Union of Pure and Applied Chemistry.^54^ They have a high surface area, pore volume, and well-organized mesoporous texture.^55,56^ MBG was first synthesized by Yan et al. in 2004 by combining the sol-gel process with the supramolecular chemistry of surfactants. The glass powder obtained had well-ordered mesoporous channels in the range of 4–7 nm (hexagonal symmetry of pore arrangement).^57^ The emergence of these glass powders has opened up new opportunities in biomaterials research. Over the past two decades, MBGs have been extensively utilized in various applications within regenerative medicine. They have proven effective in supporting bone regeneration and treating bone diseases through the controlled release of drugs, inorganic ions, and organic compounds directly at the injured site.^58^ MBGs offer a versatile platform for targeted therapies and have shown promise in bone tissue regeneration.^56,59–63^

Inorganic therapeutic agents can be incorporated into CaPs, BGs, and MBGs to ensure extra functions, such as osteogenesis and angiogenesis, as well as antibacterial and anti-inflammatory activities. For example, the most reported metal ions are silver (Ag^+^), lithium (Li^+^), copper (Cu^2+^), strontium (Sr^2+^), cobalt (Co^2+^), zinc (Zn^+2^), magnesium (Mg^2+^), gallium (Ga^3+^) and cerium (Ce^3+^ and Ce^4+^).^55,56,64,65^

Sánchez-Salcedo et al. produced MBG scaffolds doped with silver nanoparticles (Ag_NPs). The 3D-printed scaffolds showed antimicrobial activity against *S. aureus* and *E. coli*, and the amount of Ag_NPs added to the scaffolds did not compromise cell viability.^66^ Anand et al. prepared MBG powders co-doped with Cu^2+^ and Mg^2+^ ions. They verified that co-doped MBGs showed cell proliferation and viability of osteoblast-like MG-63 cells similar to the basic glass 80S and antibacterial activity against *S. aureus* (Gram-positive) and *E. coli* (Gram-negative) bacteria.^67^

Overall, several compositions of BGs are available for clinical applications, with more than 25 BG medical devices approved worldwide.^68^ They are commercialized in different particle sizes, morphology, and compositions, allowing various applications.^68,69^ NovaBone^®^, for instance, offers several 45S5 BG-based bone grafts for dental and orthopedic uses.^70,71^ BonAlive^®^ provides S53P4 BG both as granules and paste to act in the treatment of bone infections, benign bone tumors, and spine or trauma surgeries.^70^

### Calcium silicates (Ca-Si)

Calcium silicates (Ca-Si) are a class of bioceramics broadly spread in dentistry and bone tissue engineering. Their biological outcome relies on releasing calcium and silicon ions in situ. While calcium ions are crucial in mediating metabolic responses on bone regeneration,^72,73^ silicon ions aid in the early stages of calcification, cell attachment, and angiogenesis.^74–76^ Significantly, their properties vary according to their composition, divided into monocalcium silicate (CaSiO_3_), dicalcium silicate (Ca_2_SiO_4_), tricalcium silicate (Ca_3_Si2O_7_), and pyrosilicate (Ca_3_Si_2_O_7_).^77^

Wollastonite is one of the most relevant Ca-Si for bone regeneration. It consists of a monocalcium silicate with a structure divided into α-wollastonite (monoclinic), stable at temperatures superior to 1120 °C, and β-wollastonite, which is stable at lower temperatures and can be found either in a monoclinic or triclinic structure, with 6 polytypes depending on the number of subcells.^78,79^ Overall, wollastonite is a bioactive and osteoconductive ceramic with high mechanical resistance,^80–82^ which makes it a promising material for scaffold manufacturing.

Ca-Si phases are the main components of mineral trioxide aggregate (MTA) cement, whose clinical applications include pulp capping,^83,84^ root-end filling,^84,85^ and perforation repair.^84,86^ By controlling the setting time, these cements can produce 3D-printed ceramic grafts^53^ or improve composite scaffolds’ printability and biological response.^87,88^ In addition, doping Ca-Si might be a promising way to enhance their biological response. For instance, incorporating Sr in Ca-Si scaffolds showed good results both in vitro and in vivo, aiding osteoblastic differentiation, reducing osteoclastogenesis, improving angiogenesis, and increasing mechanical resistance.^89,90^ Chen et al. reported that 3D-printed lithium Ca-Si scaffolds present dual bioactivity, acting on subchondral bone and cartilage regeneration in rabbit models, which is promising to repair osteochondral defects.^91^ Du et al. compared Mg^2+^ and Mn^2+^ ions as doping agents on calcium silicate used to produce scaffolds by the sol-gel technique. Both showed improved bone-marrow stem cells (BMSCs) differentiation, vascularization, and new bone formation, with Mg^2+^ presenting slightly better results in vitro and in vivo.^92^ It is worth mentioning that several other doping agents have been reported, such as copper,^93^ gadolinium,^94^ zinc,^95^ and lanthanum.^96^

### Emerging and specialized ceramics

The number of bioceramics reported for bone regeneration is extensive. Several other classes have been explored with exciting responses. For instance, magnesium phosphates (MgP) are newly developed resorbable ceramics with a higher dissolution rate than CaPs.^97^ Silicon nitride (Si_3_N_4_) stands out among other bioceramics for its good mechanical response and osteogenic and antibacterial properties.^98–100^ Piezoelectric ceramics are also attractive due to the activation of unique pathways for bone regeneration through the emission of electric signals. Barium titanate, for instance, is a piezoelectric ceramic often combined with other bioceramics, such as HA,^101,102^ β-TCP,^103^ and BGs,^104^ aiming to amplify their regenerative outcomes. Thus, there is a plethora of ceramics to explore, and more products are expected to reach the market in the coming years.

### Composites (Polymers/Ceramics)

3D printing of polymer-ceramic composites holds immense potential in craniomaxillofacial bone reconstruction and repair.^105^ There are some key reasons why it is crucial in craniomaxillofacial bone applications.^105^ Composite ceramics 3D printing enables the creation of highly customized implants tailored to each patient’s anatomy.^105^ These materials are biocompatible and integrate well with the patient’s bone tissue, reducing the risk of rejection or allergic reactions. Maxillofacial implants made from composite ceramics are durable and can withstand the daily forces applied during chewing, speaking, and facial expressions.^105^ Composite ceramics are lightweight materials with low Young’s moduli, making them ideal for craniomaxillofacial applications.^105^ Lightweight and flexibility ensure that the implant does not apply load into the surrounding tissues and bones, promoting patient comfort and reducing the risk of complications. Notably, these materials exhibit high stiffness compared to pure ceramic scaffolds, making them suitable for load-bearing sites. Furthermore, in craniomaxillofacial reconstruction, aesthetics is often as important as functionality.^105^

Composite TCP-based scaffolds with different fiber laydown patterns, coated with hydroxyapatite, were used to optimize bone regeneration in critical-sized calvaria defects in the rat skull. The composite scaffolds had a diameter of 5 mm and a thickness of 2 mm and were composed of 80 wt% PCL and 20 wt% TCP, with a porosity of 67%–71%, a pore size of 420–500 µm, and a surface area of 65–73 mm^2^. The 0°/60°/120° laydown pattern resulted in superior bone formation and biomechanical properties compared to the perpendicular 0°/90° pattern in all experimental groups.^106^

GelMA/nHAp microgel arrays were created by combining GelMA solutions of varying weights (5% and 10% w/v) with nHAp at different concentrations (1%, 2%, and 3% w/v). Scanning electron microscopy images revealed that elevating the nHAp concentration resulted in a reduction in pore size within the hydrogel and an augmentation in the roughness of the micropore walls. This suggests that incorporating higher levels of nHAp imparts distinct morphological characteristics to the microgel arrays.^107^

In an in vivo study, three scaffolds with varying pore sizes (350, 500, and 800 µm) and unique architecture were implanted in immunocompromised mice after seeding with bone morphogenetic protein-7-transduced human gingival fibroblasts. The pore size of the composite (PCL/HA) scaffolds did not affect bone regeneration in vivo.^108^ In another study, Lee et al. 3D printed PCL/β-TCP scaffolds with heterogeneous pore sizes and wing-like structures promoted greater bone formation in a large critical-sized defect in the mandible of dogs,^109^ highlighting the importance of the pore size for bone regeneration. Considering the wide range of materials that can be used to obtain bone grafts, selecting the most appropriate technique according to the targeted reconstructed area is essential in the regenerative context.

## Manufacturing technologies for fabricating bioceramic grafts – conventional vs. 3D printing

Multiple techniques have been documented for producing bioactive and resorbable ceramic scaffolds, which can be categorized into conventional and AM methods. Among the traditional methods are the porogenic agent,^110,111^ the foam replication technique,^112–115^ gel casting of foams,^116–118^ freeze casting,^119,120^ and their combination.^121,122^

Conventional techniques face difficulties in achieving precise control over parameters such as porosity, interconnectivity of pores, and structural characteristics (e.g., pore size and shape).^123^ As a result, ensuring the reproducibility of the graft design is challenging.^124,125^ Moreover, one of the main challenges of conventional methods is the inability to develop scaffolds or grafts that match the complex anatomy of the patient’s defects. As a result, a notable amount of material waste is generated, making this technique unsustainable. On the other hand, AM techniques, generically referred to as 3D printing, emerge as an alternative to meet the desired requirements of scaffolds. These techniques allow the development of anatomically complex and personalized geometries while offering high levels of process control and reproducibility, taking bone graft production to a higher level.^124–127^

Generally, AM techniques consist of printing objects via layer-by-layer deposition of powder, liquid, or solid materials from a computer-aided designed (CAD) model. This results in significant loss reduction, process flexibility, logistics facilitation, free-form fabrication, and patient-specific fidelity.^125,128^ This technology was first developed by Charles Hull in 1986 in a process known as stereolithography (SLA) and has since evolved with the emergence of new methodologies, materials, and printing techniques.^128^

According to the International Organization for Standardization (ISO) and the American Society for Testing and Materials (ASTM), AM technologies can be classified into several categories: (1) vat photopolymerization, (2) material extrusion, (3) material jetting, (4) binder jetting, (5) powder bed fusion, (6) sheet lamination, and (7) direct energy deposition.^19^ Herein, a particular focus will be on the material extrusion technique since it is one of the most widely applied techniques for craniomaxillofacial bone regeneration.

In recent years, numerous pre-clinical studies have been reported on using bioceramics scaffolds in different animal models,^129,130^ including 3D-printed scaffolds. However, their clinical applications remain considerably behind the extensive reports on bone tissue engineering research. There are two main obstacles in the translation from research to clinic. Firstly, the poor mechanical performance of bioceramics, especially in terms of tensile strength, highly limits most clinical applications.^131,132^ In addition, there needs to be a clear correlation between the ideal bioceramics graft (i.e., composition and overall porosity) and the defect type (i.e., type of bone, defect anatomical location, and its dimension).^131,133^ Thus, there is a clear need for a better understanding of the scaffold’s features and production and the correlation between in vitro and in vivo models with specific bone types and defects.

## Material-based extrusion AM—Direct-ink writing (DIW)

**Fig. 2:**
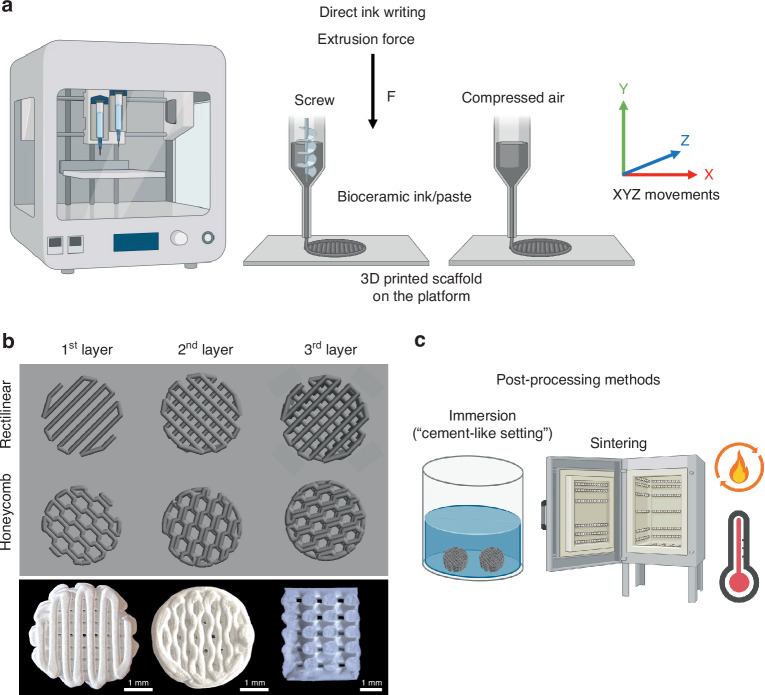
Schematic of DIW for printing bioceramics. **a** Printing process of scaffolds either by extrusion screw or compressed air methods; **b** Scaffolds’ design according to different rectilinear or honeycomb infilling patterns layer-by-layer, top view and cross-section of scaffolds after printing; **c** Post-processing methods for bioceramic scaffolds after printing. Created using Biorender.com

Among the 3D printing technologies available, material extrusion, also known as direct ink writing (DIW) or robocasting, stands out in producing bioceramic parts, so a particular focus will be given to this technique. DIW allows the manufacture of pieces of various sizes, with great versatility in raw material selection, relatively low production costs, excellent resolution of printing (between 5 and 200 μm), and high printing speed.^128^ This 3D printing technique allows the fabrication of scaffolds by stacking layers of ceramic materials in a grid-like structure.^134^ In this process, the layers are produced by extruding ceramic paste or ink through a nozzle that can move in three dimensions (Fig. 2a). The movement of the nozzle is programmed to create scaffolds with a structure designed computationally. With the precise control offered by the 3D printer, highly accurate scaffold replicas of the desired model can be obtained (Fig. 2b). This level of control ensures a high degree of accuracy in determining the scaffold’s final porosity and pore shape.

To successfully manufacture ceramic scaffolds by DIW, the ceramic ink should present adequate rheological properties. According to the literature, to achieve optimal material extrusion and the formation of continuous filaments without clogging, the ink used in the process should exhibit shear-thinning behavior and recovery after extrusion. This property enables the ink to flow easily through the nozzle during extrusion and regain its shape afterward, ensuring the structural integrity of the printed object.^135,136^

There are several other crucial aspects to consider when utilizing ceramic ink in 3D printing,^20^ including: (i) Particle Size: The particle size of ceramic materials should typically range between 1 and 10 µm. This enables the production of ceramic ink with a high solid content, typically around 40–50 vol.% or 60–80 wt%^137^; (ii) Printing Speed: The printing speed needs to be carefully adjusted to avoid the generation of defects in the printed part. Proper printing speed control is vital to ensure optimal print quality and structural integrity^127,135,138^; (iii) Nozzle Diameter and Printing Design: The diameter of the printing nozzle and the overall printing design plays a significant role in determining the accuracy and resolution of the printed structures^139^; (iv) Drying and Sintering Parameters: Parameters related to drying and sintering should be carefully optimized. These parameters affect the elimination of the organic phase of the material and promote densification during the sintering process, ultimately influencing the final properties of the printed ceramic structure^140^; (v) Choice and Amount of Additives: The selection and quantity of additives in ink are crucial for ensuring fluidity within the printing channel and cohesion of the printed structure. Additives are essential to maintain the ink’s desired properties and enable successful printing.^126,127,138^

The printed scaffolds, called green bodies, usually need to go through a post-treatment to consolidate their structure. When producing crystalline ceramic scaffolds, the green bodies are submitted to a heat treatment that includes calcination and sintering steps, in which the organic materials are eliminated, and the microstructure is consolidated through mass diffusion between the ceramic particles. Each ceramic has its ideal sintering temperature, roughly between 50% and 75% of its melting point.^141^ It is essential to remember that some ceramics undergo phase transformation during the heat treatment, which can affect their performance.

BGs are non-crystalline materials that can crystallize upon sintering. When crystallization occurs, and it is intentional, a glass-ceramic with a similar composition to the precursor glass but with different properties is achieved.^142,143^ Another example is β-TCP (rhombohedral, *ρ* = 3.07 g/cm^3^), which can present the formation of the α-TCP phase (monoclinic, *ρ* = 2.86 g/cm^3^) upon sintering. This phase transformation may lead to the formation of microcracks on the printed object due to the difference in density between the two phases,^29,144,145^ and thus decrease its mechanical resistance.

Another consolidation technique employed after printing by DIW is cross-linking the polymeric structure. This process does not eliminate the organic matter, resulting in composite scaffolds. Thus, the polymer used in the ink formulation must be biocompatible. Compared to the sintering process, polymer cross-linking allows the incorporation of drugs directly into the ink,^146^ in addition to avoiding phase formation and transformations of the ceramic, which can, for instance, preserve the non-crystalline structure of BG scaffolds.^147^ Each polymer has its cross-linking mechanism and requires a different approach after printing. For example, sodium alginate (Na-alg) cross-links its structure by replacing the sodium in its composition with Ca^2+^, Al^3+^, or Mg^2+^ ions, forming a chemical bond between different polymeric chains.^148^ Another study reported the comparison of scaffolds after sintering and cross-linking their structure by printing hydroxyapatite/Na-alg scaffolds. The sintered HA scaffolds presented higher mechanical resistance and lower porosity, of 9.5 MPa and 44%, than the cross-linked HA/Na-alg scaffolds, of 2.6 MPa and 74%.^149^

Other techniques for fabricating robust structures include using cement’s self-setting properties. The cement setting time is strictly controlled during printing, and the setting reaction is responsible for the scaffold’s structural hardening. The most common cement precursors in 3D printing for bone regeneration are tricalcium phosphates,^140^ monetite, brushite, and calcium silicate.^150^ After printing, the scaffold is dipped in a solution to complete the setting reaction^151^ (Fig. 2c).

The printing and post-processing steps may directly affect the scaffold’s design since the particle size, printing speed, needle diameter, post-processing methods, and the presence of additives might tamper with the scaffold’s geometry regarding pore size or shape. Consequently, design plays an important role in the interaction between the cells and the bone graft, and these properties will be deeply explored in the following section.

## Design impact on craniomaxillofacial bone regeneration

The scaffold’s design properties affect biological properties such as cell adhesion, proliferation, and osteogenic differentiation of stem and progenitor cells. 3D printing technology allows for precise control over scaffold design and fabrication, making it an ideal method for producing patient-specific scaffolds.

**Fig. 3:**
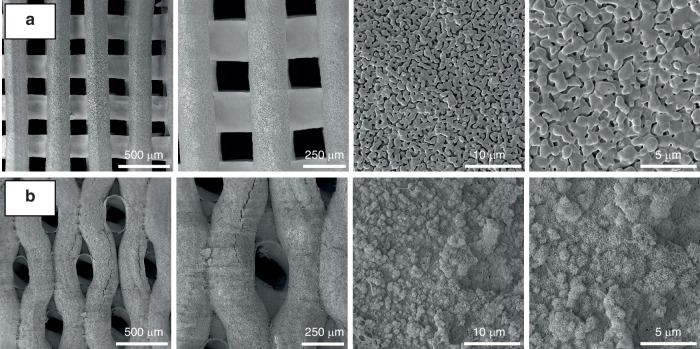
SEM images of 3D printed bioceramic scaffolds according to different designs and materials. **a** β-TCP in a rectilinear infilling pattern, evidencing the square-shaped pores and microstructure; and (**b**) Calcium phosphate cement (Osteoink^TM^) in a honeycomb infilling pattern, presenting hexagonal-shaped pores and microstructure. Magnifications of 85×, 150×, 5k×, and 10k×, respectively

This section discusses the impact of the 3D-printed scaffold’s geometry, pore size, surface area, and pore morphology on craniomaxillofacial bone regeneration. Various aspects of porosity, such as the quantity, dimensions, morphology, and arrangement, must be considered (Fig. 3).

### Geometry

3D printing technology allows for creating intricate structures that accurately mimic the macro/microgeometry of native bone tissue.^152^ Of note, extensive bone reconstructions and personalization of bone grafts are two significant challenges in regenerative dental medicine. To address this issue, Anderson et al. utilized β-TCP and hydroxyapatite/α-TCP (Osteoink^TM^) inks to print, based on cone beam computed tomography (CBCT) imaging data, personalized bioceramic grafts.^13^ Hayashi et al. 3D printed scaffolds with different shapes, including dense granules (DGs) and two variations of honeycomb (HCGs), characterized by the presence or absence of protuberances, each measuring 75 μm in length.^153^ Notably, adding protuberances increased the granule’s surface area by approximately 3.24 mm^2^. However, this improvement also led to a wider gap between the scaffolds, resulting in an overall increase in the space within the defect by approximately 7.6%.^153^ In the case of DGs, the formation of new bone occurred exclusively on the granule’s surface. Conversely, HCGs exhibited a distinct behavior, with bone formation co-occurring on both the surface and within the intra-scaffold channels. HCGs lacking protuberances demonstrated the formation of roughly 30% more new bone compared to their protuberance-containing counterparts.^153^ These findings underscore the significant influence of the shape and geometry of scaffolds on bone formation, considering relevant factors such as the impact of increasing the surface area of the scaffold.

### Surface modification

The biological interactions of implantable biomaterials with natural tissues depend significantly on the material’s surface properties. Alterations to surface topography, roughness, or chemistry can result in optimal performance regarding osseointegration, biocompatibility, and mechanical strength.^154^ There are two categories of surface modifications: physical and chemical. Chemical changes involve coating or chemical grafting, while physical modifications alter the surface’s topography, roughness, and morphology. Both categories present interesting results according to the literature, so the clinical context and targeted area to be reconstructed should be considered when these modifications are applied to bone grafts. Orthopedic surgeons face challenging cases in bone regeneration, such as spinal fusion and long bone fractures, and craniomaxillofacial surgery is even more complex due to the proximity of bones to nerves. Our discussion encompassed various surface modifications and their implications in bone regeneration.

#### Physical modifications

Modifying material surfaces with nanotopography enhances cell adhesion and can influence cell differentiation, improving osseointegration, biocompatibility, and mechanical resistance. Further, bioactive components are widely used to modify surface roughness and bioactivity. For example, mesoporous silica nanoparticles have been utilized as a nanocarrier due to their desirable characteristics, including their large surface area, well-defined pore structure, excellent biocompatibility, and easy functionalization.^155^ In an in vitro and in vivo study, Zhang and colleagues utilized 3D printing to create a magnesium scaffold with an adjustable pore structure, which was further surface-modified with a calcium phosphate coating.^156^ Adding calcium phosphate onto material surfaces enhanced their biocompatibility and biosafety. In addition, including Mg^2+^ during synthesis enhances the thermal stability of HA. It leads to a more stable phase composition after heat treatment, allowing for the creation of porous or granulated scaffolds for various biomedical applications, such as orthopedics and craniomaxillofacial surgery.^157^

Furthermore, calcium phosphate coatings can promote osteogenesis and angiogenesis. The vascularization process heavily relies on the interaction between endothelial and smooth muscle cells and the scaffold surface, particularly in the initial stage. Research has demonstrated the influential role of surface topography, encompassing features such as grooves, pitches, and curves, in regulating vasculogenesis. The deliberate engineering of surfaces to possess a tailored micro-nanoscale topographic milieu, informed by biomimetic principles, has been observed to benefit vasculogenic activity.^158^ Interestingly, by adjusting the thickness of the coating, it is also possible to control the degradation rate of the materials effectively. This provides a means to fine-tune the biodegradation properties and tailor the material’s performance for specific applications. ^159^

#### Chemical modifications

In addition to physical modifications, chemical treatments of scaffold materials with cell-specific ligands can facilitate cell adhesion and regulate biological functions. This is particularly important for rapid vascularization. Amino acid sequences, including Arg-Gly-Asp (RGD), Tyr-Ile-Gly-Ser-Arg (YIGSR), and Ile-Lys-Val-Ala-Val (IKVAV), have been extensively studied for their ability to enhance endothelial cell adhesion through the establishment of ligand-modified surfaces.^160^ Hao et al. aimed to discover new ligands and identified an αvβ3 integrin ligand called LXW7. The ligand was developed using unnatural amino acids and demonstrated better stability, binding affinity, and specificity when compared to peptides composed of only natural amino acids.^161^

Polydopamine coatings have gained significant attention for improving the properties of materials in various fields, including medicine.^162^ Lee and colleagues developed a technique to create versatile polymer coatings on numerous materials by dip-coating them in an aqueous dopamine solution. They took inspiration from the composition of mussel adhesive proteins. Dopamine self-polymerization generated thin, surface-adherent polydopamine films on a wide range of inorganic and organic materials, such as noble metals, oxides, polymers, semiconductors, and ceramics. These films can undergo secondary reactions to form a variety of ad-layers, including self-assembled monolayers via deposition of long-chain molecular building blocks, metal films through electroless metallization, and bioinert and bioactive surfaces by grafting of macromolecules.^163^

### Macro-, micro-, and nanoporosity of printed structures

The formation of the extracellular matrix, the infiltration of blood vessels, and the exchange of nutrients and waste materials are contingent upon the scaffold’s geometric characteristics, specifically the pore architecture. While solid (bulk) β-TCP constructs can undergo gradual absorption, their slow degradation rate constrains their applicability in a developing skeletal context, where grafts must adapt and remodel per the patient’s growth.^164^ In the context of growth factor carriers and promoting bone repair, the porosity of a scaffold (particularly interconnected porosity) is also considered more critical than its composition. This interconnected porosity enables effective entrapment and retention of circulating growth factors, essential for stimulating and supporting bone repair, thus promoting efficient bone regeneration.^165^ In 3D printing, strand distance, and fiber alignment/orientation are crucial for forming and integrating new bone tissue with the host bone. The struts’ architectural variation affects the scaffolds’ surface volume and permeability. For instance, scaffolds with a 0°/90° fiber alignment displayed significantly greater porosity and larger pore size while exhibiting a lower scaffold surface area compared to those with a 0°/60° fiber alignment.^106^

Research has shown that scaffolds’ microstructure, such as the size of pores and porosity of scaffolds, impact bone regeneration.^166,167^ Entezari et al. used 3D printing to create bioceramic scaffolds with varying porosities and pore structures. The findings demonstrated that scaffolds possessing a pore size of approximately 390 μm displayed superior in vivo bone formation. Conversely, when the pore size exceeded 590 μm, changes in scaffold pore size did not result in more significant new bone formation.^168^

In bone tissue engineering, the mean pore size of a scaffold is a crucial factor that plays a decisive role in determining the extent of blood vessel ingrowth. The pores’ size directly influences the blood vessels’ ability to penetrate and infiltrate the scaffold, promoting vascularization within the engineered tissue. The design of the scaffold significantly affects the vascularization rate after implantation. Comparing cylindrical types of different diameters, new bone and bone marrow formation was observed to be more significant in the larger ones (300 and 500 μm) than the smaller ones (50–100 μm) in a porous hydroxyapatite material.^169^ Implants with a pore size (565 μm) demonstrated superior bone formation in both peripheral and deep pores than implants with smaller pore sizes (300 μm). However, no significant differences were observed between implants with 40% and 50% macroporosity. This suggests that the size of the macropores had a more substantial impact on bone ingrowth than the percentage of microporosity.^169^

A critical consideration in scaffold design is striking a balance between porosity, which aids in mass transport for biological delivery and tissue regeneration, and the mechanical properties required for temporary scaffold function. To address this challenge, Hollister proposed an approach that accurately determines the mechanical and mass transport properties at a specific scale, considering detailed properties and structure.^170^ Achieving hierarchical design can be facilitated by developing libraries of unit cells, which are mathematical constructs rather than biological cells, at different physical scales. These unit cells can then be assembled to create scaffold architectures that meet the desired mechanical and transport requirements, enabling more effective regenerative strategies.^171^ Nonetheless, there is still no consensus in the literature about the optimal pore size that meets all the requirements to support an ideal bone formation, so further studies are needed. However, two points can be confidently asserted. First, producing a scaffold material with small, monomodal pores is not advisable. Research suggests that scaffolds with pore diameters below 100 μm may hinder the delivery of oxygen and nutrients to the scaffold’s core, as cells tend to adhere and accumulate on the scaffold’s surface, obstructing the pores.^172^ Another significant point is that a biomimetic-graded porous structure comprising both macropores and micropores provides the most favorable conditions for angiogenesis. Mimicking the natural tissue’s architecture, this graded porous structure facilitates the formation of blood vessels within the scaffold. The combination of macropores allows for the infiltration of endothelial cells and the establishment of a vascular network. At the same time, the presence of micropores promotes cell adhesion and the diffusion of nutrients and oxygen throughout the scaffold. This biomimetic approach offers a promising strategy to enhance vasculogenesis and support the successful integration of engineered tissues with the host vasculature. However, determining the appropriate pore size for scaffolds in craniomaxillofacial bone regeneration remains debatable. A recent review by Marques et al. suggested that 96–150 μm micropores could promote osteoblasts migration and protein adhesion. In contrast, macropores larger than 300 μm may be more suitable for bone and capillary growth.^173^ Another work showed that scaffold pore sizes ranging from 500 to 600 μm provided the most favorable conditions for osteoblast adhesion to the surface of biomaterials.

### Interconnectivity

In addition to pore size, interconnectivity is a critical factor that affects the body and bioceramic scaffold interaction. Interconnected pores within a scaffold create a pathway for cells to migrate and transport nutrients.^174^ The scaffold’s internal 3D and interconnected pore structure serves a dual function – facilitating the exchange of nutrients and metabolites and guiding cell growth. Furthermore, the scaffold possesses sufficient strength to meet the mechanical requirements of the implantation site during the early stages.^175,176^ Recently, Gu et al. utilized Mg-doped tricalcium phosphate to fabricate a 3D-printed scaffold with interconnected pores with mechanical properties comparable to bone.^177^ The scaffold was seeded with bone marrow and umbilical cord-derived mesenchymal stem cells (MSCs), which enhanced osteogenesis and angiogenesis in vitro. Kim et al. successfully repaired a maxillary tumor defect in a dog using 3D-printed scaffolds with complete interconnectivity composed of β-TCP-based paste.^178^ According to Liu et al., a research investigation revealed that porous scaffolds exhibiting interconnectivity and possessing a pore size of 500 μm displayed optimal osteogenic differentiation of human bone marrow-derived mesenchymal stem cells in vitro and notably enhanced cell proliferation at a 60% porosity level.^179^ Moreover, introducing extra struts diminished the interconnectivity between pores, potentially impacting bone formation to some degree.^180^ This finding aligns with prior studies, which reported an initial increase in bone regeneration rate followed by a gradual decrease after reaching a specific value for pore size.^181^

Micro-computed tomography (μCT) findings demonstrated that a bioceramic scaffold with 600 μm pores exhibited the highest volume of new bone formation and optimal trabecular number within the 2–12 weeks compared to the other groups with pore sizes of 480 and 720 μm.^182^ This finding was further supported by histological analysis, which confirmed effective new bone formation for this group of scaffolds.^182^ The observed superior outcome can be attributed to the relatively small pore size of the scaffold, which facilitated the transport of oxygen and nutrients critical for early-stage implantation. The smaller pore size likely created a favorable microenvironment that supported cell viability, proliferation, differentiation, and subsequent bone tissue deposition. Although all three scaffold groups had the same porosity, the group with 720 μm pores had less canal structure and interconnectivity than the other groups with smaller pore sizes, resulting in limited space for new bone tissue growth.^182^ Researchers also have proposed a hypothesis that increasing the pore dimension to a certain extent may result in saturation of nutrient and oxygen supply, leading to a plateau in the osteogenic effect.^182^

Understanding the intricacies of bone formation necessitates the utilization of diverse animal models, each offering unique advantages that contribute to a holistic understanding of this process. Particularly, when assessing critical size defects, the suitability of animal models, such as rats and larger species, is paramount. Additionally, distinctions between load-bearing and non-load-bearing defects are crucial. Load-bearing defects necessitate more stringent evaluation, as the mechanical environment significantly influences bone healing. When evaluating a rat critical-sized calvarial defect, it was observed that nanocomposite scaffolds made of polydopamine-laced hydroxyapatite collagen calcium silicate (HCCS-PDA) with a high degree of porosity and larger pore size (500 μm) exhibited remarkable bone regeneration in comparison to scaffolds with a lower degree of porosity and smaller pore size (250 μm). The HCCS-PDA scaffolds, with their higher porosity, provided an optimal environment for crucial factors such as cell infiltration, vascularization, and nutrient exchange, which played vital roles in promoting more efficient bone regeneration. The larger pore size likely facilitated better cell migration, adhesion, and the deposition of essential extracellular matrix components, ultimately contributing to superior bone formation in the critical-sized defect.^183,184^

In contrast, an alternative study conducted in a rat critical-sized calvarial model revealed that a 3D-printed β-TCP scaffold with a pore size of 100 μm exhibited the highest percentage of new bone ingrowth, surpassing scaffolds with larger pore sizes of 250 μm and 400 μm. These findings indicate that the smaller pore size of the β-TCP scaffold promoted more favorable conditions for bone ingrowth. The 100 μm pore size likely facilitated better cell attachment, proliferation, and the establishment of a well-integrated network of new bone tissue within the critical-sized defect.^185^ These results highlight that further investigation and comprehensive understanding are needed to elucidate the relationship between porosity and pore interconnectivity to optimize scaffold design.

### Shapes of pores

**Fig. 4:**
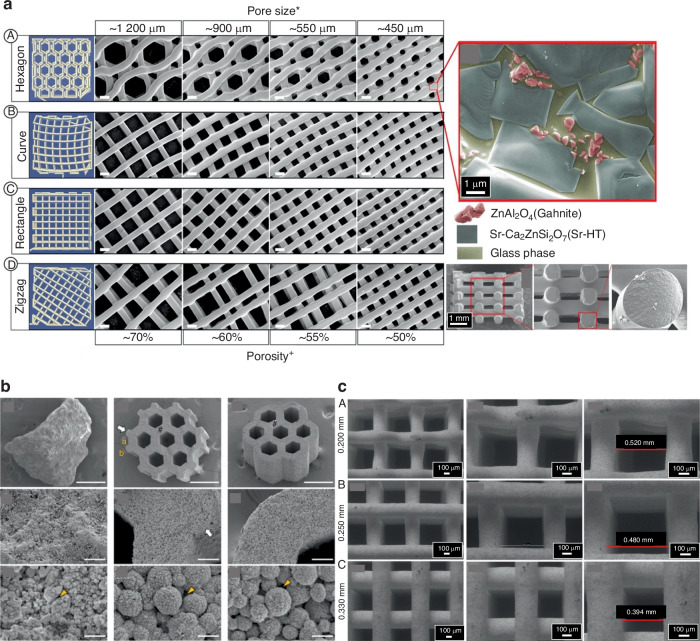
Computer design and SEM images according to different designs, pore sizes, and porosities of Sr-HT-Gahnite scaffolds prepared by DIW (**a**); SEM images of dense granules, scaffolds with or without protrusions which might increase their surface area (**b**); and SEM images of β-TCP scaffolds printed with different needle internal diameters and spacing between filaments (**c**). Figures **a** and **b** were adapted from refs. ^138^ and^153^, respectively (open-access articles distributed under the terms of the Creative Commons CC BY license). Figure **c** was adapted from.^277^

The shape of pores in scaffolds plays a crucial role in bone regeneration. Studies have shown that different pore shapes, such as cylindrical, spherical, or irregular, can influence cell behavior, nutrient diffusion, and vascularization within the scaffold^186^ (Fig. 4). Pore shape affects cell attachment, alignment, and migration, ultimately impacting tissue ingrowth and the formation of new bone. Additionally, specific pore shapes can promote the infiltration and organization of vasculature, facilitating the delivery of oxygen and nutrients to support cellular activity.^186^ Therefore, the appropriate pore shape is essential for designing scaffolds that promote optimal bone regeneration and tissue integration. Wu et al. showed that the gyroid-pore structure exhibited accelerated ion dissolution and mass degradation during in vitro testing.^186^ When tested in rabbit models, it was challenging to achieve efficient vascularization even in the 650-μm pore region of hexagon-pore scaffolds within 2 weeks.

In contrast, gyroid-pore scaffolds displayed significant blood vessel networks after 2 weeks, even in the 350 μm pore region. Furthermore, after 4 weeks, high-density blood vessels uniformly penetrated the 500 and 650 μm pores of gyroid-pore scaffolds. Cube-pore scaffolds also demonstrated a higher capacity to promote angiogenesis within four weeks than hexagon-pore scaffolds.^186^

Barba et al. reported that the spherical, concave macropores present in foamed scaffolds made of β-TCP exhibited superior efficacy in promoting material resorption and bone regeneration, surpassing the performance of 3D-printed scaffolds with orthogonal-patterned struts and thus prismatic, convex macropores. This observation suggests that the specific geometry of the macropores significantly influences the biological response and subsequent tissue regeneration. The spherical, concave macropores likely provide a more favorable microenvironment for cellular activity, facilitating efficient material resorption and promoting enhanced bone regeneration within the scaffold.^175,187^ Scaffolds with a cross-shaped pore structure may exhibit inferior performance in strength and osteoconductivity. Therefore, using porous HA with cylindrical pores offers distinct advantages and may be preferred over materials with alternative pore structures for bone regeneration.^162^ According to Bidan et al., their findings indicate that in the case of cross-shaped pores with a non-convex structure, the initial overall tissue deposition occurs twice as fast as square-shaped pores with a convex structure. This suggests that the specific pore geometry plays a significant role in the rate of tissue deposition.^188^ Square and star geometries are considered less favorable for craniomaxillofacial bone regeneration. This assessment is based on limited cell attachment and spreading, reduced nutrient diffusion, and compromised vascularization within scaffolds with square and star-shaped pores in vivo. These geometries may hinder optimal cellular behavior, impede tissue ingrowth, and adversely affect the overall regenerative capacity of the scaffold.^189^

Li et al. devised a novel 3D-printed bioceramic scaffold inspired by the hot dog structure, featuring drug loading and cell transport capabilities. Utilizing extrusion 3D printing and the two-way ice template method, they created a bioceramic rod with a hollow tube (resembling the bread in a hot dog, with a 1 mm tube diameter) and a sausage-like shape (resembling the sausage, with a 500 μm diameter). The sausage structure exhibited an organized and uniform layered micropore arrangement with an average diameter of around 30 μm. The hollow tube structure of the scaffold facilitated the growth of blood vessels and the formation of new bone.^190^

Korn et al. devised a personalized geometry of the bone defects, employing two distinct pore designs: a 60-degree and a 30-degree rotated layer orientation.^191^ These scaffolds were fabricated using an extrusion-based 3D printing technique with calcium phosphate cement. Subsequently, the scaffolds were implanted into artificial bone defects created in the palates of mature Lewis rats, and they exhibited robust structural support.^191^ Their findings highlight a significant impact on the pore geometry, with the 60-degree orientation outperforming the 30-degree counterpart. However, pre-colonization of the scaffolds with rMSCs did not amplify bone healing.^191^

## Personalized grafts for craniomaxillofacial applications

**Fig. 5:**
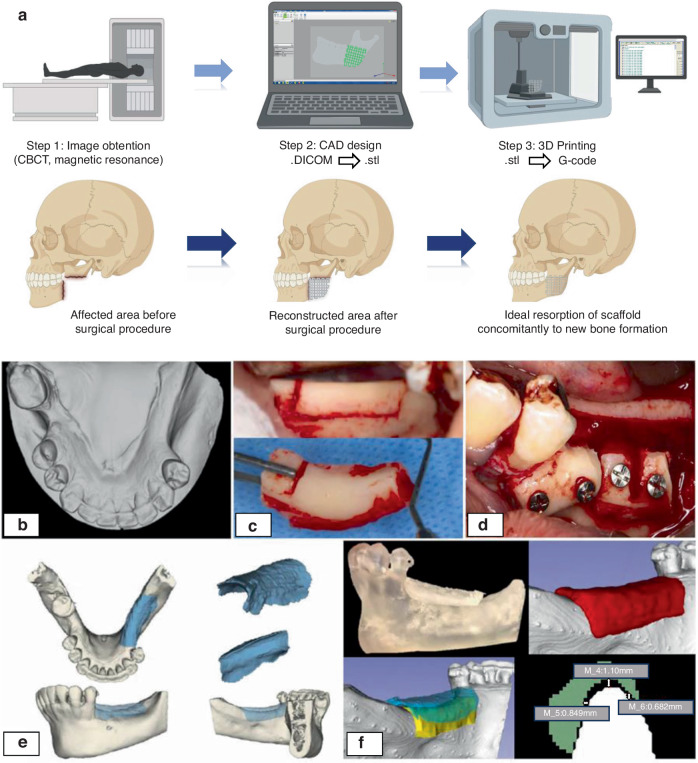
The workflow process of 3D printed personalized grafts for maxillofacial reconstruction. **a** From image obtention by imaging exams, design on CAD software, and printing process by G-code file (created using Biorender.com) of the personalized graft. **b**–**f** A clinical procedure involving mandibular bone reconstruction using autologous bone harvested from left ramus. A 3D-printed bioceramic graft was used for a comparison purpose, in which the printed bioceramic graft was scanned to ensure the perfect match between the area of interest and the internal surface of the graft (adapted from Anderson et al.^13^)

Following an extensive discussion about the critical factors concerning bone grafts, covering bioceramic materials, AM techniques focusing on DIW particularities, and the impact of the graft’s design on bone regeneration, a particular focus is given to engineering personalized grafts. One of the most significant advantages of applying 3D printing for craniomaxillofacial applications is the possibility of producing personalized grafts that perfectly fit the bone defect.^10^ Combining imaging examinations, computer-aided design (CAD) planning, and additive manufacturing makes it possible to develop personalized grafts matching craniomaxillofacial bone defects (Fig. 5a).^9^ Besides the benefits of producing patient-specific geometries, the possibility of having bone grafts ready for placement in the region of interest before the surgical procedure might reduce the surgical time,^13^ promoting a faster process and reducing patient trauma.

Cone-beam computerized tomography (CBCT) is frequently used to obtain high-resolution image stacks, exported in Digital Imaging and Communications in Medicine, also known as.DICOM files. Magnetic resonance imaging is also a viable alternative to provide high-quality images, especially for reconstructing cartilage zones.^192^ As a first step, the images are combined, forming a 3D model of the area to be recovered,^13,193,194^ in which the morphology and limits of the region/volume of interest can be precisely identified in a three-dimensional view. Thus, a slicer software is used to convert the DICOM files into 3D models and correct the model, removing artifacts or unwanted images and consequently improving the model’s visualization.^13^ The obtained 3D models will be generated in standard tessellation language (.stl) file format, creating a mesh composed of vertices and edge-sharing triangular shapes.^164,193^

After this process, the scaffold is designed according to the desired shape and size of the region of interest. When designing the scaffold’s shape, it is essential to match the outer edge of the defect to the newly created inner edge of the scaffold. Thus, after checking the surfaces, it is recommended to rescale the model to reduce its size by a factor of 0.98 to ensure it will fit the patient’s region of interest perfectly.^10^ If this reduction is not performed, the inner edge of the scaffold will be precisely the same size as the outer edge of the defect, resulting in an over-contour or poor adaptation, for example. All the specifications are defined in this step, including the shape and limits of the scaffold, the presence or absence of pores, pore size, and geometry.^13,164^ Further CAD processing steps might be required for both 3D models (area of interest and designed scaffold), such as hole filling, noise reduction, cropping of useless areas, and smoothing surfaces.^10^ The .stl file is later transferred to a.gcode file,^164,193^ converting the design information mentioned before and the X, Y, and Z axis coordinates required for printing the 3D structure into codes and numbers. After converting the files, the 3D printing process is ready to start, and the manufacturing software provides the working time and remaining time for printing. When printing, the cartridges of the 3D printer will follow the coordinates generated by the.gcode extruding the selected material.^164^

Confirming the clinically suitable adaptation of patient-specific grafts, Anderson et al. evaluated the precision fit of printed scaffolds compared to their respective defect areas using 3D models obtained from CBCT and image scanning (Fig. 5b–f), which were superimposed with the best matching algorithm method.^13^ After aligning the models of the defective area and the 3D printed scaffolds, the average fit deviation was just 0.27 mm, evidencing a high level of precision and clinically accepted adaptation of the printed scaffolds, regardless of the defect morphology or size.^13^

Besides suitable adaptation, good stability was also observed by Schulz et al., who evaluated the clinical application of 3D printed calcium phosphate cement scaffolds for sinus grafting, considering the scaffold’s placement and posterior implant surgery rehabilitation for maxillary bone. The scaffolds were obtained by combining CBCT images, CAD software, and 3D printing. Nine months after scaffold placement, implants were installed to reestablish the patient’s dentition. Satisfactory primary stability of the implants was evidenced during surgery, and no abnormalities or postoperative complications were observed.^12^

The demand for personalized 3D grafts is expected to grow rapidly by 2030,^195,196^ creating opportunities for new companies and products to develop. However, the translation of personalized grafts into the clinics is still a challenge, both technically and in terms of administrative aspects. Firstly, there is a need to integrate grafts into clinical procedures. The overwhelming publications on bone research literature need to transition to clinical practice due to a lack of an adequate link between the features and composition of the graft with the specific bone and defect types.^131,133^ Besides tailoring design and material to specific clinical needs, major administrative and regulatory challenges must be overcome, including financial, insurance, and legal aspects.^195,196^ Specifically for personalized grafts, the legislation needs to be clarified, and advances in bone research must be followed.^197^

A deep understanding of the legislation and regulatory protocols can be a differential factor for the company’s success and product consolidation in the market. In the United States, the Food and Drug Administration (FDA) released in 2017 the overall guidelines for Additive Manufactured Products, followed by the creation of an Emerging Technology Team (ETT) responsible for identifying and resolving regulatory hurdles on the application of emerging manufacturing in the biomedical market.^198–200^ Across the world, several regulations on using 3D-printed personalized devices have been created recently.^201^ However, these regulations are still recent, and new companies must establish a workflow with more precise and clear guidelines, covering all the steps from the patient diagnosis to the personalization of the graft.

## In vitro evaluation of 3D printed scaffolds

**Fig. 6:**
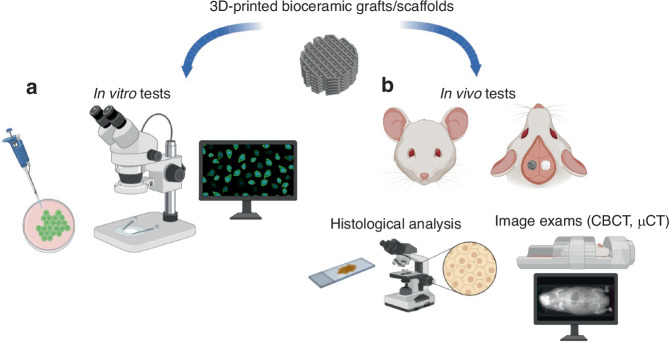
Outcomes assessment for bioceramics scaffolds after printing (**a**) In vitro and (**b**) in vivo analysis. The figure was created using Biorender.com

In vitro assays are the first methodology to evaluate biomaterials since they can analyze the interaction between the materials and cells in a controlled laboratory setting (Fig. 6a).^202^

**Fig. 7:**
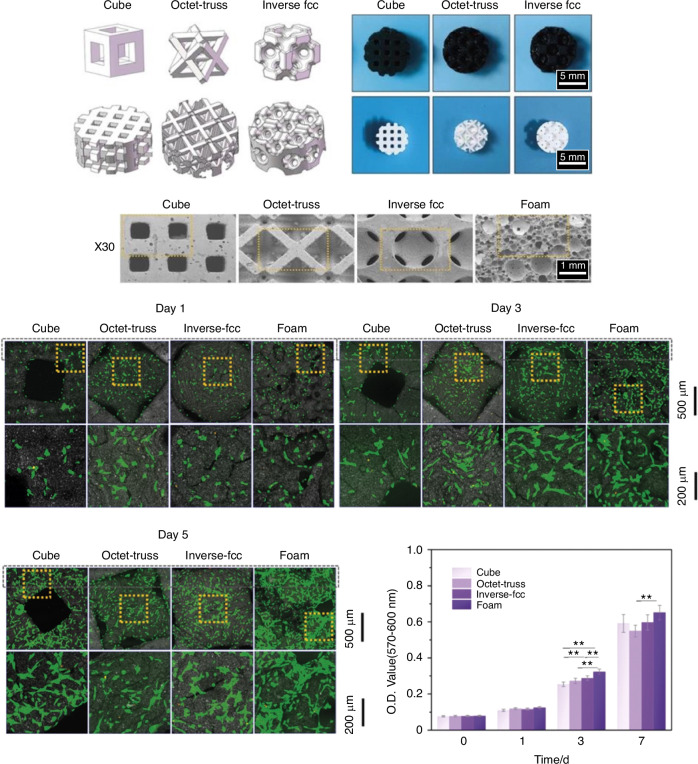
Routine in vitro assessment for 3D printed bioceramic scaffolds. Scaffolds’ design on CAD software and overall morphology after printing via SEM; cells attached to the scaffolds under confocal laser scanning microscopy and cell viability analysis (AlamarBlue assay). Figure adapted from Wang et al.^278^ Open access article distributed under the terms and conditions of the Creative Commons Attribution (CC BY) license (https://creativecommons.org/licenses/by/4.0/)

The starting point is to provide a biocompatible scaffold that shows no- or low-level toxicity.^11^ Also, it has already been reported that macro and micro geometries of the scaffolds present great importance on cell penetration and adhesion, processes that mediate osseointegration.^203,204^ These processes are crucial for promoting osseointegration, i.e., the successful integration of the scaffold with the surrounding bone. The macro geometry of the scaffold, including its overall shape and architecture, influences factors such as load distribution and mechanical stability, which are essential for proper cell migration and tissue formation discussed previously. The microgeometry, including pore size, interconnectivity, and surface roughness, directly impacts cell adhesion, migration, and nutrient diffusion within the scaffold (Fig. 7). By carefully controlling scaffolds’ macro and micro geometries, cell penetration and adhesion can be enhanced, improving osseointegration and tissue regeneration outcomes. MSCs are the most common cell type to evaluate the scaffold’s bioactivity potential since they can differentiate into several cell types, such as bone (osteoblasts) or adipose (adipocytes) cells.^6^ Thus, cell viability, alkaline phosphatase activity assay (ALP), and the expression of osteogenesis-related genes are the most used in vitro tests to evaluate the biological potential of scaffolds for bone tissue engineering.^205^

It is well-established that the natural release of ions, such as Ca^2+^ and PO_4_^3−^, from calcium phosphate bioceramics can effectively guide cell migration and promote the formation of a mineralized matrix. These ions play a crucial role in regulating cellular activities. They are involved in critical processes related to bone regeneration, including cell adhesion, proliferation, differentiation, and the deposition of mineralized tissue. The controlled release of these ions provides a favorable microenvironment for cellular interactions and facilitates the successful integration of the scaffold with the surrounding tissues.^206^ Anderson et al. analyzed two ceramic materials (hybrid calcium phosphate and Osteoink^TM^).^13^ They found that the cell viability was significantly reduced when the bone marrow cells were exposed to the hybrid calcium phosphate. This fact can be due to the pH of this material, which decreased rapidly over 24 h compared to Osteoink^TM^. Therefore, it shows that certain changes in the bone environment can be harmful to cells.^13^

Besides the effects of bioceramics on the cells, the materials incorporated in the ceramics and released after the scaffold’s application also play an important role in cell behavior, such as ions or bioglass particles. For instance, bioactive ions such as Ca^2+^, Si^4+^, PO_4_^3-^, Fe^2+^, Cu^1+^, and Se^2-^ incorporated into a ceramic material promoted osteogenesis of bone marrow-derived MSCs in vitro.^203,207^

Another factor that can influence the cell’s behavior is the design of the printed scaffolds since the architecture will define the properties, nutrient transport, and the interaction between the scaffold and the cell matrix.^203^ A previous study, which analyzed the osteogenesis potential of carbonate apatite scaffolds with different shapes (honeycomb design with or without protrusions), showed that the honeycomb design increased cell proliferation, alkaline phosphatase activity, and mineralized nodules formation when compared to dense granules.^153^ Another design modification that presented promising results was the hollow-pipe structure, which differs from the regular scaffolds, showing a ring-shaped cross-section. This structure resulted in a higher surface area for cell adhesion and proliferation, enhanced vascularization, and accelerated scaffold degradation, responsible for releasing ionic products into the environment.^208^

Altogether, in vitro assays provide valuable insights into the biocompatibility and bioactivity of biomaterials, helping to screen and select promising candidates for further investigation. Nonetheless, it is paramount to understand the signaling pathways and their effect on cellular response/behavior when investigating these bioceramics.

### Signaling pathways

The energy metabolism of osteoblasts is critically dependent on mitochondrial activity, which supplies the ATP necessary for various cellular processes, including bone matrix synthesis and mineralization.^209^ Ceramic materials and 3D graft structures can significantly influence the metabolic activity of osteoblasts, thereby affecting bone formation.^210^ Studies have shown that bioceramics, such as hydroxyapatite and tricalcium phosphate, can enhance osteoblast function due to their bioactive properties, which promote cell adhesion, proliferation, and differentiation.^211^ These materials’ surface characteristics and porosity are crucial, as they can modulate the cellular microenvironment, impacting nutrient diffusion and waste removal, essential for mitochondrial function.^212^

Furthermore, 3D-printed graft structures with controlled macroporosity and interconnected pore networks provide a scaffold that mimics the extracellular matrix, facilitating better cell infiltration and vascularization.^212^ This structural mimicry supports enhanced nutrient and oxygen supply to osteoblasts, optimizing their metabolic activity and ensuring an adequate energy supply for bone formation. Additionally, the mechanical properties of 3D-printed bioceramic grafts can influence cellular mechanotransduction pathways, further modulating mitochondrial activity and energy production.^213,214^ These combined effects underscore the importance of material composition and architecture in designing bone grafts that not only support osteoblast viability but also enhance their metabolic efficiency, thereby promoting effective bone regeneration.^215^

This section delves into the intricate signaling pathways intertwined with the fascinating realm of major bioceramics. Exploring the molecular cues and regulatory networks, we uncover the dynamic interplay between bioceramic materials and cellular responses (Table 2).

**Table 2:** Concise signaling pathway information of main bioceramics, including hydroxyapatite, bioactive glasses, and calcium phosphates.^237^

Aspect	Hydroxyapatite	Bioactive Glasses	Tricalcium Phosphate (TCP)	Calcium Silicate	Calcium Phosphate Cement
Composition	Ca_10_(PO_4_)_6_(OH)_2_	Various compositions including Si, Ca, Na, P	Ca_3_(PO_4_)_2_	CaO-SiO_2_-H_2_O system	Ca_5_(PO_4_)_3_OH and CaHPO_4_
Biocompatibility	High	High	High	High	High
Osteoconductivity	Excellent	Excellent	Excellent	Excellent	Excellent
Signaling Pathways	Wnt/β-catenin pathway	Surface Ion Exchange and HA Layer Formation	BMP Pathway	Notch Signaling	Wnt/β-catenin Pathway
	BMP Pathway	RANKL/RANK/OPG pathway	Wnt/β-catenin Pathway	Hedgehog Pathway	BMP Pathway
	Hedgehog Pathway	Hedgehog Pathway	Notch Signaling	RANK/RANKL/OPG Pathway	ERK Pathway
	Notch Signaling	--	ERK Pathway	--	--
	RANK/RANKL/OPG Pathway	--	Angiogenic Signaling Pathways	--	--
Biological Effects	Bone mineralization and remodeling	Enhanced osteogenic differentiation	Osteogenic differentiation and bone formation	Angiogenesis	Bone mineralization and remodeling
	Dental enamel and dentin structure	Angiogenesis and tissue regeneration	Angiogenesis	Immune response modulation	Mechanical strength and rigidity of bone tissue
	Mechanical strength and rigidity of bone tissue	Immune response modulation	Inflammatory response modulation	--	--

#### Calcium phosphates

Although calcium phosphates (CaPs) do not possess intrinsic signaling properties like some growth factors or cytokines, their interaction with biological systems can modulate cellular signaling pathways involved in bone formation, remodeling, and tissue regeneration. Calcium and phosphate signaling mediate numerous downstream signaling cascades in diverse cell populations.^216,217^ Below, we provide a concise overview of the major signaling pathways associated with CaPs.

##### Osteogenic differentiation pathways

Research indicates that CaP facilitates the process of osteogenic differentiation in mesenchymal stem cells (MSCs) and osteoblasts through various signaling pathways, including:

- Bone Morphogenetic Protein (BMP) Pathway: CaP can upregulate the expression of BMPs and BMP receptors, leading to activation of the canonical BMP signaling pathway. This pathway involves the phosphorylation and nuclear translocation of Smad proteins, which regulate the transcription of genes involved in osteogenic differentiation and bone formation.^218^ Adding calcium ions to cell culture media has been shown to increase the expression of bone morphogenetic protein-2 (BMP-2) as well as other markers associated with osteogenesis, such as Osteocalcin (OCN), Osteopontin (OPN) and Collagen Type I (COL-I), in both Mesenchymal Stem Cells (MSCs) and bone cells.^219^ The upregulation of BMP-2 expression appears to be linked to the MEK1/2 pathway activation through calcium channels, facilitating MSC differentiation.^220^ Phosphate can activate cAMP signaling and induce BMP-2 expression and secretion.^221^ Within osteoclasts, phosphate signaling acts in opposition to RANK-RANKL signaling, indicating the presence of a negative feedback loop during the process of bone resorption.^222^ Phosphate also influences the expression of osteopontin (OPN) via PKC and ERK1/2 signaling.^223,224^ Calcium and phosphate exhibit comparable effects on genes associated with osteogenesis, such as BMP-2, highlighting their importance in bone regeneration.^224^
- Wnt/β-catenin Pathway: CaP can trigger Wnt signaling, which results in the stabilization and movement of β-catenin into the nucleus. Once in the nucleus, β-catenin governs the expression of specific genes crucial for osteoblast differentiation and the formation of bone.^35^ This pathway plays a crucial role in osteogenesis, the process of new bone formation. Upon binding of Wnt ligands to cell surface receptors, a series of signaling events is initiated, culminating in the stabilization and buildup of β-catenin within the cytoplasm. Subsequently, the accumulated β-catenin translocates to the nucleus, where it engages with transcription factors like TCF/LEF, prompting the transcription of genes crucial for osteoblast differentiation and the formation of bone.^225^
- Notch Signaling: CaP can modulate Notch signaling, which plays a role in cell fate determination and osteogenic differentiation. Activation of the Notch pathway can influence the balance between osteoblastogenesis and adipogenesis in MSCs.^226^
- Extracellular Signal-Regulated Kinase (ERK) Pathway: CaP can activate the ERK signaling pathway, which regulates cell proliferation, survival, and differentiation. ERK activation has been associated with enhanced osteogenic differentiation of MSCs and osteoblasts. Calcium signaling initiates the Mitogen-Activated Protein Kinase (MAPK) pathway, akin to integrin signaling. Extracellular calcium triggers the expression of Cyclooxygenase-2 (COX-2) in osteoblasts through the Protein Kinase A (PKA) pathway. This induction leads to the activation of ERK1/2 signaling, subsequently impacting osteoblast differentiation.^227^ Phosphate signaling also influences osteoblasts, osteocytes, and osteoclasts. Phosphate controls Matrix Gla Protein (MGP) activity in osteoblasts via ERK1/2 phosphorylation.^228^
- Myosin light chain kinase (MLCK): Calcium ions bind to various effector proteins, such as calmodulin, which then regulate downstream cellular processes. For example, the calcium-calmodulin complex can activate enzymes like protein kinase C (PKC) or myosin light chain kinase (MLCK), leading to changes in cell function.^229^

##### Angiogenic Signaling Pathways

CaP can also influence angiogenesis, the process of new blood vessel formation, through various signaling pathways,^230^ including:

- Vascular Endothelial Growth Factor (VEGF) Pathway: CaP can increase the expression of Vascular Endothelial Growth Factor (VEGF), a powerful angiogenic factor, in osteoblasts and various other cell types. VEGF promotes endothelial cell proliferation, migration, and the formation of tubes, thereby facilitating angiogenesis.
- Hypoxia-Inducible Factor (HIF) Pathway: CaP can stabilize and activate HIF-1α, a transcription factor involved in cellular responses to hypoxia. Hypoxia-inducible factor 1 alpha (HIF-1α) controls the expression of genes associated with angiogenesis, including Vascular Endothelial Growth Factor (VEGF).

- Fibroblast Growth Factor (FGF) Pathway: CaP can induce the release of FGFs from osteoblasts and other cell types. FGFs stimulate endothelial cell proliferation and migration, contributing to angiogenesis.

##### Inflammatory and Immune Response

CaP implants can trigger an inflammatory reaction, marked by the attraction of immune cells like macrophages and neutrophils to the implantation site. The interaction between CaP and immune cells can modulate inflammatory signaling pathways,^231^ such as:

- Nuclear Factor-kappa B (NF-κB) Pathway: CaP can stimulate NF-κB signaling within immune cells, generating pro-inflammatory cytokines and chemokines. NF-κB activation can also influence osteogenic differentiation and bone formation in MSCs and osteoblasts.
- Interleukin-6 (IL-6) Signaling: CaP can induce the production of IL-6, a pleiotropic cytokine involved in inflammation and bone metabolism. IL-6 can stimulate osteoblast differentiation and inhibit osteoclast formation, contributing to bone regeneration.

#### Bioactive glasses

Bioactive Glass (BG) influences the function of numerous genes crucial for cell growth, specialization, and the development of bone tissue. Particularly noteworthy are alkaline phosphatase (ALP), bone sialoprotein (BSP), Runt-related transcription factor 2 (Runx2), and osteocalcin (OCN).^232,233^ These glasses typically contain elements such as silicon (SiO^2^), calcium (Ca^2+^), sodium (Na^+^), and phosphorus (PO_4_^3−^), among others. The signaling pathways associated with bioactive glasses involve a complex interplay between the material’s surface properties, the surrounding biological environment, and the cells interacting with the material. The next section summarizes the major signaling pathways associated with BGs.

- Surface Ion Exchange and Formation of Hydroxyapatite (HA) Layer: Upon contact with physiological fluids, bioactive glasses undergo surface reactions, releasing ions such as Ca^2+^, Si^4+^, and PO_4_^3−^. These ions can modulate various cellular processes by activating specific signaling pathways. For example, the release of Ca^2+^ ions can trigger signaling cascades involved in cell adhesion, migration, and differentiation. The release of Si^4+^ ions has been shown to stimulate the expression of osteogenic genes and promote osteoblastic differentiation.^234^ Additionally, the formation of a hydroxyapatite layer on the glass surface provides a bioactive interface for cell attachment and activation of signaling pathways associated with bone formation. Moreover, bioactive glasses can impact the RANKL/RANK/OPG system, potentially hindering osteoclastogenesis while fostering bone formation. For instance, studies demonstrate that strontium-substituted bioactive glasses inhibit osteoclastogenesis by dampening RANKL-induced signaling pathways, while lithium ions released from certain bioactive glasses can similarly inhibit osteoclastogenesis.^235^
- Hedgehog pathway: The Hedgehog (HH) pathway represents another significant signaling pathway involved in bone development. Ligands such as Sonic Hedgehog (SHH) and Indian Hedgehog (IHH) play pivotal roles in regulating this process.^236^ Preliminary evidence suggests that bioactive glasses may influence HH pathways, with ions like strontium and lithium potentially affecting these pathways.^236^ Nevertheless, additional research is required to fully understand the intricate mechanisms underlying the interaction between bioactive materials and these signaling pathways and their implications for bone biology.
- Activation of Integrin-Mediated Signaling: Cell adhesion to bioactive glass surfaces is primarily mediated by integrin receptors, which interact with specific ligands on the material surface. Engagement of integrins initiates intracellular signaling pathways, including focal adhesion kinase (FAK) and Src kinase signaling, which regulate cytoskeletal organization, cell spreading, and survival.^237^ These pathways can activate downstream signaling cascades, including the mitogen-activated protein kinase (MAPK) pathway and the phosphoinositide 3-kinase (PI3K)/Akt pathway, impacting cell behavior and fate.^237^ The Mitogen-Activated Protein Kinase (MAPK) family, consisting of extracellular signal-regulated protein kinases (ERK), p38, c-Jun N-terminal kinase (JNK), and ERK5, is instrumental in governing cellular processes, including differentiation, proliferation, apoptosis, and response to stress.^238,239^ Various studies have observed changes in MAPK gene expression when cells are cultured with BG.^240,241^ Additionally, high concentrations of silicon (SiO_2_) and calcium (Ca^2+^) can activate MAPKs, which are also essential components of BG’s bioactivity.^240^ Nevertheless, the specific mechanism through which Bioactive Glass (BG) modulates gene expression via the MAPK pathway remains uncertain.
  Moreover, ions such as Mn^2+^, Ca^2+^, Zn^2+^, Sr^2+^, SiO_2_, Cu^2+^, Co^2+^, Li^+^, and B have been shown to influence cell function and subsequent bone formation when released from biomaterials.^242^ These ions may impact various pathways and cell types, but interpreting their effects is challenging due to the complex release dynamics and potential cytotoxicity at certain levels. Calcium ions, particularly from calcium phosphate-based biomaterials, are noteworthy in this context, as their release can modulate cell phenotype and downstream signaling pathways, including MAPK, cAMP-PKA, and PI3K-AKT, ultimately influencing osteoblast differentiation.^242^
- Release of Growth Factors and Cytokines: Bioactive glasses have been shown to adsorb and release various growth factors and cytokines in the surrounding biological environment. For example, Bioactive glasses can adsorb bone morphogenetic proteins (BMPs), insulin-like growth factors (IGFs), and transforming growth factor-beta (TGF-β), all of which play pivotal roles in the process of bone regeneration and repair.^237^ The release of these bioactive molecules from the glass surface can activate specific receptor-mediated signaling pathways in nearby cells. This adsorption ultimately enhances osteogenic differentiation, angiogenesis, and tissue regeneration.
- Inflammatory and Immune Response: Following implantation, bioactive glasses can trigger an inflammatory response marked by the immune cells’ recruitment, including macrophages and neutrophils, to the implantation site. These immune cells release various cytokines and chemokines that modulate the local microenvironment and activate signaling pathways involved in tissue repair and regeneration. For instance, the secretion of pro-inflammatory cytokines like tumor necrosis factor-alpha (TNF-α) and interleukin-1 (IL-1) can initiate downstream signaling pathways, including nuclear factor-kappa B (NF-κB) and MAPK pathways. These pathways govern cell proliferation, differentiation, and the resolution of inflammation.^243^

#### Hydroxyapatite

Hydroxyapatite (HA) is a naturally occurring mineral form of calcium apatite. While hydroxyapatite itself is not directly involved in signaling pathways, its presence and interaction with biological systems can influence various cellular signaling pathways through a process known as biomineralization. Biomineralization is the process by which living organisms produce minerals, such as hydroxyapatite, within their tissues. In the context of bone formation and remodeling, hydroxyapatite deposition and dissolution are tightly regulated processes mediated by various signaling pathways involving cells such as osteoblasts, osteoclasts, and osteocytes. In the next section, we provide a concise overview of the major signaling pathways associated with HA.

##### Osteoblast differentiation

Osteoblasts are bone-forming cells responsible for synthesizing and mineralizing the bone matrix, which includes hydroxyapatite. Signaling pathways like the BMP pathway are pivotal in guiding the differentiation of mesenchymal stem cells into osteoblasts.^244^ BMPs are growth factors classified within the transforming growth factor-beta (TGF-β) superfamily, pivotal for orchestrating osteoblast differentiation and bone formation processes.^244^ BMP signaling initiates with the binding of BMP ligands to cell surface receptors, which triggers downstream signaling cascades such as the Smad pathway. Combining HA with other materials, such as gold, promoted catenin-mediated osteogenesis in MSCs.^245^ Additionally, Bone Morphogenetic Protein (BMP) signaling synergizes with Wnt signaling to regulate osteoblast differentiation, particularly through canonical signals.^246^ Studies have revealed increased expression of osteogenic genes and BMP2/Smad signaling pathway components in MSCs cultured with HA, highlighting the intricate interplay between HA, Wnt, and BMP pathways in osteogenesis.^247^

- Extracellular Matrix Production: Osteoblasts secrete extracellular matrix proteins like collagen, which provide a scaffold for mineral deposition. Signaling pathways such as Wnt/β-catenin are involved in regulating osteoblast activity and extracellular matrix production.^244^ The Wnt signaling pathway is essential for cell determination, proliferation, and differentiation. It functions through interactions between Wnt proteins and Frizzled (FZD) receptors, notably the low-density lipoprotein receptor-related protein 5/6 (LRP5/6) coreceptors. Activation of the Wnt pathway results in the stabilization and migration of β-catenin to the nucleus following Wnt-FZD-LRP5/6 binding. Within the nucleus, β-catenin controls the expression of genes critical for osteoblast differentiation and bone formation.^248^ Functionally, Wnt/-catenin signaling is pivotal in promoting osteoblast differentiation and sustaining bone mass.^249^ Wnt10b, among various Wnt ligands, is notable for its role in osteoblast differentiation, orchestrating the expression of key transcription factors like Runx2, Dlx5, and Osx while suppressing adipogenic factors C/EBP_ and PPAR.^244,250^ The interplay between Wnt signaling and osteogenesis is evident in the presence of HA.^244^ Furthermore, the morphological characteristics of HA particles influence Wnt signaling activation, as demonstrated by enhanced osteogenic differentiation of MSCs on strontium-doped HA-coated surfaces with nanorod-patterned features.^244,251^
- Mineralization Initiation: Nucleation of HA crystals begins within the collagen matrix. Signaling molecules like osteopontin and osteocalcin help nucleate hydroxyapatite crystals and regulate their growth.^244^
- Matrix Vesicle Formation: Osteoblasts secrete matrix vesicles, small membrane-bound vesicles containing calcium and phosphate ions. These matrix vesicles provide a microenvironment conducive to hydroxyapatite crystal formation. Signaling pathways like the TGF-β (Transforming Growth Factor-beta) pathway regulate matrix vesicle formation.^244^
- Hydroxyapatite Crystal Growth and Maturation: Calcium and phosphate ions form HA crystals within the matrix vesicles, which grow and mature within the extracellular matrix. Various signaling molecules, including alkaline phosphatase, control the mineralization process by regulating the local concentration of calcium and phosphate ions.
- Notch Signaling and ERK pathway: Notch signaling is involved in cell fate determination and plays a role in osteoblast differentiation and bone formation. The Notch pathway’s activation is triggered by ERK pathway activity, which oversees the expression of genes implicated in osteoblast differentiation and bone matrix deposition. Diverse stimuli, including HA, can induce ERK pathway activation.^252^ The Extracellular Signal-Regulated Kinase (ERK) Signaling Pathway is a fundamental mechanism in cellular physiology. It is orchestrated by protein kinases, which promote the transfer of phosphate groups from ATP to target proteins. This process modulates the enzymatic activities of these proteins and their interactions with other molecules.^253^ Among the protein kinases, the Mitogen-Activated Protein Kinases (MAPKs) family stands out for its pivotal role in governing diverse cellular processes from proliferation to apoptosis.^253^ Studies demonstrated that the introduction of nanoHA augmented the expression of Osteopontin (OPN) while decreasing that of Alkaline Phosphatase (ALP) in Bone Marrow Stromal Cells (BMSCs) and preosteoblasts via the ERK pathway.^253^ Mechanistically, nanoHA interacts with Fibroblast Growth Factor Receptor (FGFR) and Phosphate Transporter (PiT), triggering ERK activation.^252^ Moreover, the physical characteristics of HA, such as its micro/nano flake-like structure, profoundly influence ERK signaling, gene expression, and osteogenic protein production in Mesenchymal Stem Cells (MSCs).^254^
- Hedgehog (Hh) Pathway: Hedgehog signaling is another important pathway in skeletal development and bone formation. Activation of the Hh pathway leads to the transcriptional regulation of genes involved in osteoblast differentiation and chondrocyte maturation.^244^
- Osteoclast Activation and Resorption/Bone Remodeling: Osteoclasts are responsible for bone resorption, which is essential for bone remodeling and mineral homeostasis. Signaling pathways involving RANKL (Receptor Activator of Nuclear Factor Kappa-B Ligand) and its receptor RANK regulate osteoclast differentiation and activation.^244^ This pathway regulates osteoclast differentiation and function by phosphorylating JNK, ERK, and p38 MAP kinase. Bone remodeling is a dynamic process characterized by a delicate equilibrium between bone formation by osteoblasts and bone resorption by osteoclasts. Signaling pathways like the OPG/RANKL/RANK system regulate this balance to maintain bone integrity and mineral homeostasis^244^ receptor RANK on osteoclast precursors, promoting osteoclast differentiation and activation. Additionally, osteoblasts produce Osteoprotegerin (OPG), a decoy receptor for RANK Ligand (RANKL). This interaction inhibits osteoclastogenesis and the subsequent resorption of bone tissue.^244^ Of note, surface topography plays a significant role in cellular responses to HA. Heat treatment of HA promotes a three-dimensional-like proliferation pattern in fibroblast cells, mediated by p38 activation, thus enhancing cell adhesion.^255^ Apart from its involvement in physiological events, p38 kinase plays a crucial role in osteoblast regulation by HA, mediating ECM mineralization and influencing gene expression.^244^ Additionally, p38 exhibits crosstalk with the ERK signaling pathway, collectively modulating the expression of genes essential for osteoblast differentiation.^244^

##### Angiogenic Signaling Pathways

Similar to CaP, hydroxyapatite regulates the following angiogenesis pathways.^256,257^

- VEGF Pathway: Hydroxyapatite, as a key component of CaP scaffolds, promotes angiogenesis by upregulating the expression of VEGF in osteoblasts and other cell types. This upregulation promotes endothelial cell proliferation, migration, and the formation of tubular structures, facilitating the formation of new blood vessels around the implant site. HA’s interaction with the VEGF pathway enhances vascularization, which is vital for supplying oxygen and nutrients necessary for tissue regeneration and integration of the implant.
- HIF Pathway: HA stabilizes and activates HIF-1α, a transcription factor pivotal in cellular responses to hypoxia. HIF-1α orchestrates the expression of genes crucial for angiogenesis, including VEGF. By activating the HIF pathway, hydroxyapatite promotes angiogenesis, ensuring adequate oxygen and nutrient supply to the implant site, which is essential for tissue regeneration and integration.
- FGF Pathway: HA induces the release of fibroblast growth factors (FGFs) from osteoblasts and other cell types. FGFs stimulate endothelial cell proliferation and migration, contributing to angiogenesis.

##### Inflammatory and Immune Response

- NF-κB Pathway: HA is capable of activating NF-κB signaling in immune cells, triggering the production of pro-inflammatory cytokines and chemokines. This inflammatory reaction leads to the recruitment of immune cells like macrophages and neutrophils to the implant site. Additionally, NF-κB activation influences osteogenic differentiation and bone formation in MSCs and osteoblasts, thereby contributing to tissue regeneration.^258^
- IL-6 Signaling: HA implants induce the production of IL-6, a cytokine involved in inflammation and bone metabolism. IL-6 stimulates osteoblast differentiation and inhibits osteoclast formation, promoting bone regeneration. The interplay between hydroxyapatite and IL-6 signaling plays a crucial role in modulating the inflammatory response and fostering bone healing and regeneration.^258^

Taken together, while in vitro assays and mechanistic-based experiments provide critical preliminary data, it is essential to complement them with in vivo studies to better understand the behavior of biomaterials in a more complex biological environment.

## In vivo evaluation of 3D printed scaffolds

Despite the great importance of in vitro assays, they cannot evaluate all the interactions between the biomaterial and cells because they are performed in a controlled environment and do not consider the organism as a whole, resulting in a more simplistic scenario (Fig. 6b).^202^ So, the main goal of in vitro analysis is to help screen for the best candidates for in vivo assessment or to understand mechanisms underlying cell behavior.^6^ The greatest challenge regarding in vitro and in vivo assays is to balance favorable properties when grafts are submitted to a controlled environment with isolated cells and the whole organism under load-bearing conditions.^123^

When performing an in vivo analysis, a critical-size bone defect, defined as a small intraosseous wound that will not heal by the natural osteogenesis,^194,202^ needs to be performed in animal models. Usually, for craniomaxillofacial application, the critical defect size is created in the mandible or calvaria bones,^202^ and its dimensions might vary according to the animal model or bone type.^6^ The animals can be grouped into two categories: small-animal (mouse, rat, and rabbit) and large-animal (dog, goat, pig, and sheep) models, the rationale being that small models are preferred for ethical, economic, and statistical purposes, while large models are preferred for better explaining clinical scenarios.^202,259^

Small-animal models such as mice and rabbits are the most commonly used models reported in the literature,^202^ and some studies performed tests on large-animal such as dogs.^109,175,178,187,260^ When selecting the best animal model to be used in each study, different species should be considered since the anatomical complexities between them and, consequently, some properties such as osteoinductive potential may vary according to the animal model.^164,261,262^ Carrel et al., evaluated the performance and safety of TCP/HA 3D printed scaffolds on vertical bone augmentation in a small-animal model, presenting encouraging results.^263^ In another study, the potential of PCL/β-TCP printed scaffolds and the presence of adipose stem cell aggregates were evaluated for promoting new bone formation in dog mandibular bones (Fig. 1S). The study concluded that the scaffolds seeded with adipose cells demonstrated enhanced ossification compared to those without cell seeding. This finding suggests that the presence of adipose stem cell aggregates on the printed scaffolds positively influences the bone regeneration process for mandibular repair.^260^ When conducting in vivo studies for bone tissue engineering, the scaffolds can be implanted into animals with or without the prior seeding of cells. However, the ideal scenario for biomaterials is that the new bone stimulation occurs by the scaffold itself, without the necessity of seeding of cells before the scaffold’s implantation.^6^

Two of the analyzed studies were conducted in humans.^12,13^ Anderson et al. aimed to compare hybrid calcium phosphate-based ceramic scaffolds with Osteoink^TM^ through mechanical and in vitro analysis. This study used an autogenous bone graft for in vivo bone rehabilitation instead of the experimental material, which was used just for imaging comparison. The study findings revealed that the printed scaffold demonstrated high precision, effectively matching the defect area, which highlights the exceptional precision level achievable with printed scaffolds.^13^ Schulz et al. used a 3D-printed calcium phosphate cement scaffold as an optional bone graft for sinus lift surgery, followed by implant placement in the same area. The patient’s follow-up showed that after nine months of placing the implants, a sufficient integration of the natural and new bone was observed without any sign of osteolysis or sequestration, confirming the adequate merging between the tissues and scaffolds.^12^

Image exams evaluate the scaffolds’ adaptation and new bone formation using in vivo models. Microcomputed tomography (µCT) and cone-beam computed tomography (CBCT) are the most used.^205,264^ These exams are essential for monitoring the post-surgical procedures and identifying the areas of interest, such as the defect size, when developing a patient-specific and personalized 3D-printed scaffold.^6,13^ Besides that, µCT also allows the quantification in three dimensions of new bone formation and the degree of penetration of this tissue into the scaffolds^265^ by analyzing the percentage of new bone volume over the total volume and bone mineral density according to the calcium hydroxyapatite quantification.^6^ One study used radiography to follow up on the post-surgical condition; however, this method presents some limitations since it is a 2D image evaluation.^263^

In addition to image exams, histology analysis also plays a vital role in identifying new bone formation through active osteoblasts or osteoclasts, vascularization, and degree of maturity and distribution of bone tissue.^265^ Typically, studies employ a combination of two different techniques to evaluate the presence of new bone formation.^153,265^ The first sign of bone formation identified by histological analysis is the invasion of fibrous connective tissue and capillaries on the scaffold’s pores. Then, mesenchymal cells appear near the capillaries, which differentiate into osteoblasts and gradually constitute the bone marrow cavity and osteoid, later mineralized. Lastly, the mature bone’s canals are formed with the Harvesian system present in some cases.^206^ Vascularization is very important since the tissue can undergo necrosis if there are insufficient nutrients and oxygen in the regeneration process.^6^

As a limitation of the in vivo studies, it is essential to remember that presenting single clinical cases does not refer to reality since individuals might react differently.^12^ Despite all the studies and improvements in bioengineering for the craniomaxillofacial reconstruction area, it is still challenging to predict the success of scaffolds, considering variations from patient to patient in a clinical scenario.^12,16^ Moreover, although in vivo studies can produce more reliable results compared to in vitro experimentation, there is still a need for improvements regarding standardization of the critical size defect and study design to translate the in vivo results to human clinical applications.^266^ So, further research is still needed to assess the efficacy and functionality of the printed scaffolds in human bone tissue engineering or regenerative applications.

As described previously, different factors can impact the cell’s behavior and in vivo response to the printed scaffolds. Table 3 summarizes the main articles.

**Table 3:** Overview of different studies analyzed in this review

Reference	Study goal	Materials	Printing technique	In vitro	In vivo	Main findings
Zhang et al.^208^	To produce a bioceramic containing Si, Mg and Ca ions which might induce vascularization in a hollow-pipe structure	Ca_7_MgSi_4_O_16_ and β-TCP	DIW	BMSCs and HUVECs	Small (rabbits – radius segmental defect)	Hollow-pipe structure promoted great angiogenesis and osteogenesis, enhancing vascularized bone regeneration
Lee et al.^109^	To develop a novel design of scaffolds assembling cortical and medullar bone for mandibular reconstruction of large defects	PCL and β-TCP	MHDS (DIW)	NA	Large (dogs –mandibular defect)	The novel design showed great stability and screw fixation overtime, and the combination of PCL/β-TCP showed an acceptable potential for mandible reconstruction
Golafshan et al.^274^	To develop a printed scaffold with controlled mechanical and biological properties by combining MgP bioceramic paste, Sr ions and polymer in different proportions	MgP, Sr ions and PCL	DIW	MSCs	Large (horses – hip defect)	The combinations of those components leaded to scaffolds with great mechanical and biological properties, inducing osteogenic differentiation of MSCs
Lee et al.^260^	To evaluate the ossification potential of printed scaffolds modified with bone extracellular matrix and adipose-derived stem cells	PCL and TCP	FDM (DIW)	NA	Large (dogs – mandibular defect)	The presence of adipose-derived stem cells into the scaffolds improved new bone formation without causing immune rejection
Zhang et al.^275^	To investigate potential of printed scaffolds on the drug release (RVS and SrRn) and differentiation of different types of bone cells	PCL and β-TCP	DIW	MSCs, osteoclasts and HUVECs	Small (rats – mandibular defect)	Scaffolds containing RVS/SrRn promoted in vitro and in vivo promising results, decreasing osteoclast activity and improving bone formation
Barba et al.^187^	To evaluate the importance of architecture and nanostructure of the scaffolds in the osteogenic potential	CDHA (calcium-deficient hydroxyapatite)	Robocasting (DIW)	MSCs	Large (dogs – intramuscular implantation)	Pore architecture and reactivity of the substrate directly impact bone formation
Maliha et al.^171^	To analyze the effect of 3D printed bioceramic scaffolds containing different pore dimensions and drug (dipyridamole) concentrations	β-TCP	DIW	NA	Small (rabbits – calvarial defect)	The coating of the bioceramic scaffolds with dipyridamole increased the bone growth in all tested concentrations, and small pore sizes were more favorable to bone regeneration when compared to medium or large pore sizes
Martínez-Vázquez et al.^276^	To produce a composite scaffold incorporated with antibiotic (vancomycin) to improve bone regeneration and promote drug release	HASi and gelatin	DIW	Osteoblasts	NA	The presence of gelatin in the scaffolds was beneficial to cell differentiation and gene expression, and the antibiotic was released gradually, successfully inhibiting bacteria growth
Korn et al.^191^	To analyze the effects of scaffolds pore geometry and cells seeding onto scaffolds on bone formation targeting cleft alveolar osteoplasty	CaP	DIW	MSCs	Small (rats – maxillary defect)	Pore geometry directly influenced the bone formation, however prior seeding the scaffolds with MSCs did not improved tissue regeneration and CaP cement was not degraded indicating the need of improving this material
Li et al.^190^	To develop a “hot dog-like” scaffold in a hollow tube structure with bioceramic material as an alternative to improve drug delivery approach and tissue engineering	Ca_2_MgSi_2_O_7_	DIW	BMSCs	Small (rabbits – femoral defect)	The hot dog-like structure improved the surface area of the scaffolds, being beneficial to drug delivery systems, cell proliferation and cells differentiation

Objective of the respective studies; Materials used for building the 3D printed scaffolds; printing technique; cell type used for in vitro analysis; animal model used for in vivo analysis and main findings of each study based on their conclusion

*NA* not applicable

## Conclusion and future perspectives

AM technologies are a promising alternative for the fabrication of personalized synthetic grafts/scaffolds for craniomaxillofacial bone reconstructions. In particular, material extrusion-based 3D printing stands out as a means of producing bioceramic structures quickly and affordably. Compared to conventional methods, AM technologies allow higher control over the macropore architecture, with good shape fidelity and less material waste. Moreover, they will enable the development of anatomically complex shapes and patient-personalized geometry.

This review covered how synthetic grafts’ material composition and geometry affect their biological response in vitro and in vivo. With the advancements in ink/paste formulation, process optimization, and image obtention, AM technologies are now well-positioned to generate personalized bioceramic grafts with the desired physical, mechanical, and biological properties. However, some challenges still limit the translation of synthetic bone substitutes to the clinic.

1. Vascularization Challenges: One of the critical challenges identified is the lack of vascularization in synthetic bone substitutes. This limitation is a significant barrier to synthetic grafts’ successful integration and functionality. Without adequate blood supply, the grafts may fail to support tissue regeneration and healing, leading to suboptimal clinical outcomes. Addressing vascularization issues is paramount. Incorporating bioprinting techniques to integrate vascular networks within the bioceramic graft could significantly enhance the viability and functionality of the grafts.^267^ Developing dynamic, perfusable bioreactors for pre-vascularization before implantation could be a promising approach.^268^ In addition, various 3D printing techniques, such as FRESH printing,^269^ in-foam bioprinting,^270^ and the SWIFT method,^271^ are utilized to achieve the complexity required for creating grafts. Future research should focus on developing strategies for creating vascularized grafts that mimic the natural tissue environment more closely.
2. Combination with Bioprinting: The potential of combining material extrusion with bioprinting to create grafts that mimic natural tissues, including bone and cartilage, represents a significant advancement. This approach can broaden the applications of AM in regenerative medicine, making it possible to address a broader range of reconstructive needs. The integration of bioprinting and bioceramic-based material extrusion opens new avenues for multi-tissue engineering.^267^ Research should explore the synergistic effects of combining different printing techniques and materials to develop multifunctional personalized grafts that can support the regeneration of various tissue types within the craniomaxillofacial region.
3. Translational Barriers: Despite the progress in AM technologies, several barriers still limit the clinical translation of synthetic bone substitutes. These include regulatory challenges, scalability issues, and the need for extensive preclinical and clinical validation. Collaboration between researchers, clinicians, and regulatory bodies is essential to address these translational barriers. Developing standardized protocols for fabricating, testing, and approving AM-based grafts can streamline the process and facilitate their adoption in clinical practice. In addition, integrating AI and machine learning algorithms can optimize graft design and predict biological responses, further personalizing treatment plans.^272^
4. Material Limitations: While bioceramics offer several advantages, they may only partially replicate natural bone’s complex mechanical and biological properties. Future research should explore hybrid, smart, and functional materials that combine the strengths of bioceramics with other biomaterials to enhance graft performance.^273^
5. Long-term Performance: The long-term stability and integration of AM-based grafts in the human body remain areas of concern. Extensive longitudinal studies are required to evaluate the durability and functionality of these grafts over time.

In conclusion, the advancements in AM technologies for craniomaxillofacial bone reconstructions are promising, offering significant benefits in personalization, material efficiency, and anatomical accuracy. Addressing the challenges of vascularization, material limitations, and translational barriers through interdisciplinary research and collaboration will be crucial for realizing the full potential of personalized regenerative solutions in clinical practice.

## Supplementary information

Figure1S. Supplemental Figure

Supplemental Information

## References

[1] Oryan, A., Alidadi, S., Moshiri, A. & Maffulli, N. Bone regenerative medicine: classic options, novel strategies, and future directions. *J. Orthop. Surg. Res. [Internet]* **9**, 18 (2014). 24628910 10.1186/1749-799X-9-18 PMC3995444

[2] Mahmoud, A. H. et al. Nanoscale β-TCP-Laden GelMA/PCL composite membrane for guided bone regeneration. *ACS Appl. Mater. Interfaces* **15**, 32121–32135 (2023). 37364054 10.1021/acsami.3c03059 PMC10982892

[3] Fischer, N. G., Münchow, E. A., Tamerler, C., Bottino, M. C. & Aparicio, C. Harnessing biomolecules for bioinspired dental biomaterials. *J. Mater. Chem. B.* **8**, 8713–8747 (2020). 32747882 10.1039/d0tb01456g PMC7544669

[4] Bottino, M.C. & Thomas, V. Membranes for periodontal regeneration–a materials perspective. *Front Oral Biol.* **17**, 90–100 (2015). 26201279 10.1159/000381699

[5] Aytac, Z. et al. Innovations in craniofacial bone and periodontal tissue engineering—from electrospinning to converged biofabrication. *Int. Mater. Rev. [Internet]* **67**, 347–384 (2022). 35754978 10.1080/09506608.2021.1946236 PMC9216197

[6] Dalfino, S. et al. Regeneration of critical‐sized mandibular defects using 3D‐printed composite scaffolds: a quantitative evaluation of new bone formation in in vivo studies. *Adv. Healthc. Mater [Internet]*. **15**, 12 (2023). 10.1002/adhm.202300128 PMC11469182 37186456

[7] de Souza Araújo, I. J. et al. Self-assembling peptide-laden electrospun scaffolds for guided mineralized tissue regeneration. *Dent. Mater.* **38**, 1749–1762 (2022). 36180310 10.1016/j.dental.2022.09.011 PMC9881689

[8] Dubey, N. et al. Highly tunable bioactive fiber-reinforced hydrogel for guided bone regeneration. *Acta Biomater.* **113**, 164–176 (2020). 32540497 10.1016/j.actbio.2020.06.011 PMC7482137

[9] Dubey, N., Ferreira, J. A., Malda, J., Bhaduri, S. B. & Bottino, M. C. Extracellular matrix/amorphous magnesium phosphate bioink for 3D bioprinting of craniomaxillofacial bone tissue. *ACS Appl. Mater. Interfaces* **12**, 23752–23763 (2020). 32352748 10.1021/acsami.0c05311 PMC7364626

[10] Verykokou, S., Ioannidis, C. & Angelopoulos, C. CBCT-based design of patient-specific 3D bone grafts for periodontal regeneration. *J. Clin. Med. [Internet]* **12**, 5023 (2023). 37568425 10.3390/jcm12155023 PMC10419991

[11] Wang, C. et al. 3D printing of bone tissue engineering scaffolds. *Bioact. Mater. [Internet]* **5**, 82–91 (2020). 31956737 10.1016/j.bioactmat.2020.01.004 PMC6962643

[12] Schulz, M. C. et al. Three-dimensional plotted calcium phosphate scaffolds for bone defect augmentation—a new method for regeneration. *J. Pers. Med [Internet]* **13**, 464 (2023). 36983646 10.3390/jpm13030464 PMC10058839

[13] Anderson, M. et al. Three-dimensional printing of clinical scale and personalized calcium phosphate scaffolds for alveolar bone reconstruction. *Dent. Mater. [Internet]* **38**, 529–539, (2022). 35074166 10.1016/j.dental.2021.12.141 PMC9016367

[14] Patel, S. Y., Kim, D. D. & Ghali, G. E. Maxillofacial reconstruction using vascularized fibula free flaps and endosseous implants. *Oral Maxillofac. Surg. Clin. North Am. [Internet]*. 2019 May 1 [cited 2023 Nov 4];31:259–284. http://www.ncbi.nlm.nih.gov/pubmed/30846345. 10.1016/j.coms.2018.12.005 30846345

[15] Orciani, M., Fini, M., Di Primio, R. & Mattioli-Belmonte, M. Biofabrication and bone tissue regeneration: cell source, approaches, and challenges. *Front Bioeng Biotechnol [Internet]*. (2017) Mar **23**,5. http://journal.frontiersin.org/article/10.3389/fbioe.2017.00017/full. 10.3389/fbioe.2017.00017 PMC5362636 28386538

[16] Bhumiratana, S. & Vunjak-Novakovic, G. Concise review: personalized human bone grafts for reconstructing head and face. *Stem Cells Transl. Med [Internet]* **1**, 64–69, (2012). 23197642 10.5966/sctm.2011-0020 PMC3513763

[17] Babaie, E. & Bhaduri, S. B. Fabrication aspects of porous biomaterials in orthopedic applications: a review. *ACS Biomater. Sci. Eng.* **4**, 1–39 (2018). 33418675 10.1021/acsbiomaterials.7b00615

[18] Cheng, L. et al. 3D printing of micro- and nanoscale bone substitutes: a review on technical and translational perspectives. *Int. J. Nanomed.* **16**, 4289–4319 (2021). 10.2147/IJN.S311001 PMC8239380 34211272

[19] ISO/ASTM. Standard terminology for additive manufacturing–general principles–terminology. *International Organization for Standardization*, 52900–52915 Geneva, Switzerland; 2015.

[20] Bose, S. et al. 3D printing of ceramics: advantages, challenges, applications, and perspectives. *J. Am. Ceramic Soc.* **2** (2024).

[21] Tao, O. et al. The applications of 3D printing for craniofacial tissue engineering. *Micromachines (Basel) [Internet]* **10**, 480 (2019). 31319522 10.3390/mi10070480 PMC6680740

[22] Hench, L. L., Splinter, R. J., Allen, W. C. & Greenlee, T. K. Bonding mechanisms at the interface of ceramic prosthetic materials. *J. Biomed. Mater. Res [Internet]* **5**, 117–141 (1971).

[23] Hench, L. L. Bioceramics: from concept to clinic. *J. Am. Ceram. Soc. [Internet]* **74**, 1487–1510 (1991).

[24] Baino, F., Novajra, G. & Vitale-Brovarone C. Bioceramics and scaffolds: a winning combination for tissue engineering. *Front Bioeng. Biotechnol. [Internet]*. **17**, 3 (2015). 10.3389/fbioe.2015.00202 PMC4681769 26734605

[25] Gul, H., Khan, M. & Khan, A. S. Bioceramics: types and clinical applications. In: *Handbook of Ionic Substituted Hydroxyapatites [Internet]* 53–83 (Elsevier, 2020). https://linkinghub.elsevier.com/retrieve/pii/B9780081028346000033.

[26] Kumar, A., Kargozar, S., Baino, F. & Han, S. S. Additive manufacturing methods for producing hydroxyapatite and hydroxyapatite-based composite scaffolds: a review. *Front Mater.* **17**, 6 (2019).

[27] Barrère, F., van Blitterswijk, C. A. & de Groot, K. Bone regeneration: molecular and cellular interactions with calcium phosphate ceramics. *Int. J. Nanomed. [Internet]* **1**, 317–332, (2006). PMC2426803 17717972

[28] Elliott, J. C. *Structure and chemistry of the apatites and other calcium orthophosphates* (Elsevier, 2013).

[29] Descamps, M., Hornez, J. C. & Leriche, A. Effects of powder stoichiometry on the sintering of β-tricalcium phosphate. *J. Eur. Ceram. Soc. [Internet]* **27**, 2401–2406, (2007).

[30] Chaair, H., Labjar, H. & Britel, O. Synthesis of β-tricalcium phosphate. Morphologie [Internet] **101**, 120–124, http://www.ncbi.nlm.nih.gov/pubmed/28942348 (2017). 10.1016/j.morpho.2017.06.002 28942348

[31] Carrodeguas, R. G. & De Aza, S. α-Tricalcium phosphate: synthesis, properties and biomedical applications. *Acta Biomater. [Internet]* **7**, 3536–3546, (2011). 21712105 10.1016/j.actbio.2011.06.019

[32] Dorozhkin, S. V. Calcium orthophosphates as bioceramics: state of the art. *J. Funct. Biomater. [Internet]* **1**, 22–107, (2010). 24955932 10.3390/jfb1010022 PMC4030894

[33] Bohner, M., Santoni, B. L. G. & Döbelin, N. β-tricalcium phosphate for bone substitution: synthesis and properties. *Acta Biomater. [Internet]* **113**, 23–41, (2020). 32565369 10.1016/j.actbio.2020.06.022

[34] Zhou, H., Yang, L., Gbureck, U. & Bhaduri, S. B. Sikder P. Monetite, an important calcium phosphate compound–Its synthesis, properties and applications in orthopedics. *Acta Biomater.* **127**, 41–55 (2021). 33812072 10.1016/j.actbio.2021.03.050

[35] Hou, X. et al. Calcium phosphate-based biomaterials for bone repair. *J. Funct. Biomater.* **13**, 187 (2022). 36278657 10.3390/jfb13040187 PMC9589993

[36] Litak, J. et al. Hydroxyapatite use in spine surgery—molecular and clinical aspect. *Materials* **15**, 2906 (2022). 35454598 10.3390/ma15082906 PMC9030649

[37] Funayama, T., Noguchi, H., Kumagai, H., Sato, K., Yoshioka, T. & Yamazaki, M. et al. Unidirectional porous beta-tricalcium phosphate and hydroxyapatite artificial bone: a review of experimental evaluations and clinical applications. *J. Artif. Organs* **24**, 103–110 (2021). 33893573 10.1007/s10047-021-01270-8 PMC8154753

[38] Kijartorn, P., Wongpairojpanich, J., Thammarakcharoen, F., Suwanprateeb, J. & Buranawat, B. Clinical evaluation of 3D printed nano-porous hydroxyapatite bone graft for alveolar ridge preservation: a randomized controlled trial. *J. Dent. Sci.* **17**, 194–203 (2022). 35028038 10.1016/j.jds.2021.05.003 PMC8739241

[39] Bonardi, J. P. et al. Clinical assessment of biphasic calcium phosphate in granules and paste forms in human maxillary sinus bone augmentation: a randomized, split-mouth clinical trial. *Materials* **16**, 1059 (2023). 36770066 10.3390/ma16031059 PMC9918988

[40] Richter, R. F., Ahlfeld, T., Gelinsky, M. & Lode, A. Composites consisting of calcium phosphate cements and mesoporous bioactive glasses as a 3D plottable drug delivery system. *Acta Biomater.* **156**, 146–157 (2023). 35063708 10.1016/j.actbio.2022.01.034

[41] Liu, D., Cui, C., Chen, W., Shi, J., Li, B. & Chen, S. Biodegradable cements for bone regeneration. *J. Funct. Biomater.* **14**, 134 (2023). 36976058 10.3390/jfb14030134 PMC10056236

[42] Oliveira, R. L. M. S. & Motisuke, M. Using round α-TCP granules for improving CPC injectability. *Mater. Res. Express [Internet]* **6**, 125407 (2019).

[43] Ginebra, M. P. Calcium phosphate bone cements. In: *Orthopaedic Bone Cements [Internet]*. 206–230 (Elsevier, 2008). https://linkinghub.elsevier.com/retrieve/pii/B9781845693763500101.

[44] Klammert, U., Reuther, T., Jahn, C., Kraski, B., Kübler, A. C. & Gbureck, U. Cytocompatibility of brushite and monetite cell culture scaffolds made by three-dimensional powder printing. *Acta Biomater.* **5**, 727–734 (2009). 18835228 10.1016/j.actbio.2008.08.019

[45] Castilho, M. et al. Direct 3D powder printing of biphasic calcium phosphate scaffolds for substitution of complex bone defects. *Biofabrication* **6**, 015006 (2014). 24429776 10.1088/1758-5082/6/1/015006

[46] Al-Sanabani, J. S., Madfa, A. A. & Al-Sanabani, F. A. Application of calcium phosphate materials in dentistry. *Int. J. Biomater.* **2013**, 1–12 (2013). 10.1155/2013/876132 PMC3710628 23878541

[47] Barinov, S. M. & Komlev, V. S. Calcium phosphate bone cements. *Inorg. Mater.* **47**, 1470–1485 (2011). 22332385

[48] Lukina, Y., Safronova, T., Smolentsev, D. & Toshev, O. Calcium phosphate cements as carriers of functional substances for the treatment of bone tissue. *Materials* **16**, 4017 (2023). 37297151 10.3390/ma16114017 PMC10254876

[49] Vezenkova, A. & Locs, J. Sudoku of porous, injectable calcium phosphate cements—path to osteoinductivity. *Bioact. Mater.* **17**, 109–124 (2022). 35386461 10.1016/j.bioactmat.2022.01.001 PMC8964990

[50] Cao, W. & Hench, L. L. *Bioactive Materials*. 22, (Ceramics International, 1996).

[51] Kaur, G., Pandey, O. P., Singh, K., Homa, D., Scott, B. & Pickrell, G. A review of bioactive glasses: their structure, properties, fabrication and apatite formation. *J. Biomed. Mater. Res. A* **102**, 254–274 (2014). 23468256 10.1002/jbm.a.34690

[52] Jones, J. R. Reprint of: review of bioactive glass: from Hench to hybrids. *Acta Biomater.* **23**, S53–S82 (2015). 26235346 10.1016/j.actbio.2015.07.019

[53] Arcos, D. & Vallet-Regí, M. Sol-gel silica-based biomaterials and bone tissue regeneration. *Acta Biomater. [Internet]* **6**, 2874–2888, (2010). 20152946 10.1016/j.actbio.2010.02.012

[54] Rouquerol, J. et al. Recommendations for the characterization of porous solids (Technical Report). *Pure Appl. Chem.* **66**, 1739–1758 (1994).

[55] Migneco, C., Fiume, E., Verné, E. & Baino, F. A guided walk through the world of mesoporous bioactive glasses (MBGs): fundamentals, processing, and applications. *Nanomaterials [Internet]* **10**, 2571 (2020). 33371415 10.3390/nano10122571 PMC7767440

[56] Baino, F. & Fiume, E. 3D printing of hierarchical scaffolds based on mesoporous bioactive glasses (MBGs)—fundamentals and applications. *Materials [Internet]* **13**, 1688 (2020). 32260374 10.3390/ma13071688 PMC7178684

[57] Yan, X., Yu, C., Zhou, X., Tang, J. & Zhao, D. Highly ordered mesoporous bioactive glasses with superior in vitro bone‐forming bioactivities. *Angew. Chem. Int. Ed. [Internet]* **43**, 5980–5984 (2004). 10.1002/anie.200460598 15547911

[58] Rahimnejad, M., Charbonneau, C., He, Z. & Lerouge, S. Injectable cell‐laden hybrid bioactive scaffold containing bioactive glass microspheres. *J. Biomed. Mater. Res. A* **111**, 1031–1043 (2023). 36597835 10.1002/jbm.a.37487

[59] Vallet-Regí, M. Ordered mesoporous materials in the context of drug delivery systems and bone tissue engineering. *Chemistry [Internet]* **12**, 5934–5943, (2006). 16832799 10.1002/chem.200600226

[60] Salinas, A. J., Shruti, S., Malavasi, G., Menabue, L. & Vallet-Regí, M. Substitutions of cerium, gallium and zinc in ordered mesoporous bioactive glasses. *Acta Biomater. [Internet]* **7**, 3452–3458, (2011). 21672640 10.1016/j.actbio.2011.05.033

[61] Wu, C. & Chang, J. Mesoporous bioactive glasses: structure characteristics, drug/growth factor delivery and bone regeneration application. *Interface Focus [Internet]* **2**, 292–306, (2012). 23741607 10.1098/rsfs.2011.0121 PMC3363021

[62] Wu, C. & Chang, J. Multifunctional mesoporous bioactive glasses for effective delivery of therapeutic ions and drug/growth factors. *J. Controlled Release [Internet]* **193**, 282–295, (2014). 10.1016/j.jconrel.2014.04.026 24780264

[63] Kargozar, S., Montazerian, M., Hamzehlou, S., Kim, H. W. & Baino, F. Mesoporous bioactive glasses: promising platforms for antibacterial strategies. *Acta Biomater. [Internet]* **81**, 1–19, (2018). 30273742 10.1016/j.actbio.2018.09.052

[64] Bano, S. et al. Synthesis and characterization of silver–strontium (Ag-Sr)-doped mesoporous bioactive glass nanoparticles. *Gels [Internet]* **7**, 34 (2021). 33805013 10.3390/gels7020034 PMC8103248

[65] Ciraldo, F. E. et al. Fabrication and characterization of Ag- and Ga-doped mesoporous glass-coated scaffolds based on natural marine sponges with improved mechanical properties. *J. Biomed. Mater. Res A [Internet]* **109**, 1309–1327, (2021). 33085223 10.1002/jbm.a.37123

[66] Sánchez-Salcedo, S., García, A., González-Jiménez, A. & Vallet-Regí, M. Antibacterial effect of 3D printed mesoporous bioactive glass scaffolds doped with metallic silver nanoparticles. *Acta Biomater [Internet]*. **155**, 654–666 (2023). 10.1016/j.actbio.2022.10.045 36332875

[67] Anand, A., Kaňková, H., Hájovská, Z., Galusek, D., Boccaccini, A. R. & Galusková, D. Bio-response of copper–magnesium co-substituted mesoporous bioactive glass for bone tissue regeneration. *J. Mater. Chem. B* **12**, 1875–1891 (2024). 38293829 10.1039/d3tb01568h

[68] Shearer, A., Montazerian, M., Sly, J. J., Hill, R. G. & Mauro, J. C. Trends and perspectives on the commercialization of bioactive glasses. *Acta Biomater.* **160**, 14–31 (2023). 36804821 10.1016/j.actbio.2023.02.020

[69] Cannio, M., Bellucci, D., Roether, J. A. Boccaccini, D. N. & Cannillo, V. Bioactive glass applications: a literature review of human clinical trials. *Materials* **14**, 5440 (2021). 10.3390/ma14185440 PMC8470635 34576662

[70] Jones, J. R. & Gibsonm, I. R. Ceramics, glasses, and glass-ceramics. In: *Biomaterials Science* Elsevier; 2020. 289–305.

[71] Baino, F. Ceramics for bone replacement. In: *Advances in Ceramic Biomaterials* (Elsevier, 2017). 249–278.

[72] Blair, H. C., Schlesinger, P. H., Huang, C. L. H. & Zaidi, M. Calcium signalling and calcium transport in bone disease. In: *Calcium Signalling and Disease [Internet]* (Springer Netherlands, 2007). 539–562. http://link.springer.com/10.1007/978-1-4020-6191-2_21. 10.1007/978-1-4020-6191-2_21 PMC2970924 18193652

[73] Chang, J., Zhang, X. & Dai, K. Material characteristics, surface/interface, and biological effects on the osteogenesis of bioactive materials. In: *Bioactive Materials for Bone Regeneration [Internet]*. (Elsevier, 2020). 1–103. https://linkinghub.elsevier.com/retrieve/pii/B9780128135037000017.

[74] Hung, C. J. et al. The role of integrin αv in proliferation and differentiation of human dental pulp cell response to calcium silicate cement. *J. Endod. [Internet]* **40**, 1802–1809, (2014). 25218525 10.1016/j.joen.2014.07.016

[75] Huang, S. C., Wu, B. C., Kao, C. T., Huang, T. H., Hung, C. J. & Shie, M. Y. Role of the p38 pathway in mineral trioxide aggregate-induced cell viability and angiogenesis-related proteins of dental pulp cell in vitro. *Int. Endod. J. [Internet]* **48**, 236–245, (2015). 24773073 10.1111/iej.12305

[76] Carlisle, E. M. Silicon: a requirement in bone formation independent of vitamin D1. *Calcif. Tissue Int. [Internet]* **33**, 27–34, (1981). 6257332 10.1007/BF02409409

[77] Youness, R. A., Tag El-deen, D. M. & Taha, M. A. A review on calcium silicate ceramics: properties, limitations, and solutions for their use in biomedical applications. *Silicon [Internet]* **15**, 2493–2505, (2023).

[78] Zhu, L. & Sohn, H. Y. Growth of 2M-wollastonite polycrystals by a partial melting and recrystallization process for the preparation of high-aspect-ratio particles. *J. Ceram. Sci. Technol.* **3**, 169–180 (2012).

[79] Almasri, K. A., Sidek, H. J. A. A., Matori, K. A. & Zaid, M. H. M. Effect of sintering temperature on physical, structural and optical properties of wollastonite based glass-ceramic derived from waste soda lime silica glasses. *Results Phys. [Internet]* **7**, 2242–2247, (2017).

[80] Saravanan, S., Vimalraj, S., Vairamani, M. & Selvamurugan, N. Role of mesoporous wollastonite (Calcium Silicate) in mesenchymal stem cell proliferation and osteoblast differentiation: a cellular and molecular study. *J. Biomed. Nanotechnol. [Internet]* **11**, 1124–1138, (2015). 26307836 10.1166/jbn.2015.2057

[81] Liu, X., Ding, C. & Chu, P. K. Mechanism of apatite formation on wollastonite coatings in simulated body fluids. *Biomater. [Internet]* **25**, 1755–1761, (2004). 10.1016/j.biomaterials.2003.08.024 14738838

[82] Sanmartin de Almeida, M., Fernandes, G. V., de, O., de Oliveira, A. M. & Granjeiro, J. M. Calcium silicate as a graft material for bone fractures: a systematic review. *J. Int. Med. Res. [Internet]* **46**, 2537–2548, (2018). 29848121 10.1177/0300060518770940 PMC6124267

[83] Daniele, L. Mineral Trioxide Aggregate (MTA) direct pulp capping: 10 years clinical results. *G Ital. Endod. [Internet]* **31**, 48–57, (2017).

[84] Parirokh, M., Torabinejad, M. & Dummer, P. M. H. Mineral trioxide aggregate and other bioactive endodontic cements: an updated overview – part I: vital pulp therapy. *Int Endod. J.* **51**, 177–205 (2018). 28836288 10.1111/iej.12841

[85] Lindeboom, J. A. H., Frenken, J. W. F. H., Kroon, F. H. M. & van den Akker, H. P. A comparative prospective randomized clinical study of MTA and IRM as root-end filling materials in single-rooted teeth in endodontic surgery. *Oral. Surg. Oral. Med. Oral. Pathol. Oral. Radiol. Endodontol. [Internet]* **100**, 495–500, (2005). 10.1016/j.tripleo.2005.03.027 16200680

[86] Baroudi, K. & Samir, S. Sealing ability of MTA used in perforation repair of permanent teeth; literature review. *Open Dent. J. [Internet]* **10**, 278–286, (2016). 27347231 10.2174/1874210601610010278 PMC4901194

[87] Beheshtizadeh, N. et al. 3D printing of complicated GelMA-coated Alginate/Tri-calcium silicate scaffold for accelerated bone regeneration. *Int. J. Biol. Macromol. [Internet]* **229**, 636–653, (2023). 36586652 10.1016/j.ijbiomac.2022.12.267

[88] Choi, D. et al. The effects of 3-dimensional bioprinting calcium silicate cement/methacrylated gelatin scaffold on the proliferation and differentiation of human dental pulp stem cells. *Materials [Internet]* **15**, 2170 (2022). 35329621 10.3390/ma15062170 PMC8948861

[89] Lin, K. et al. Enhanced osteoporotic bone regeneration by strontium-substituted calcium silicate bioactive ceramics. *Biomater. [Internet]* **34**, 10028–10042, (2013). 10.1016/j.biomaterials.2013.09.056 24095251

[90] Chiu, Y. C., Shie, M. Y., Lin, Y. H., Lee, A. K. X. & Chen, Y. W. Effect of strontium substitution on the physicochemical properties and bone regeneration potential of 3D printed calcium silicate scaffolds. *Int. J. Mol. Sci. [Internet]* **20**, 2729 (2019). 31163656 10.3390/ijms20112729 PMC6600364

[91] Chen, L. et al. 3D printing of a lithium-calcium-silicate crystal bioscaffold with dual bioactivities for osteochondral interface reconstruction. *Biomaterials [Internet]* **196**, 138–150, (2019). 29643002 10.1016/j.biomaterials.2018.04.005

[92] Du, Z. et al. Calcium silicate scaffolds promoting bone regeneration via the doping of Mg2+ or Mn2+ ion. *Compos B Eng. [Internet]* **190**, 107937 (2020).

[93] Mabrouk, M., ElShebiney, S. A., Kenawy, S. H., El‐Bassyouni, G. T. & Hamzawy, E. M. Novel, cost‐effective, Cu‐doped calcium silicate nanoparticles for bone fracture intervention: Inherent bioactivity and in vivo performance. *J. Biomed. Mater. Res. B Appl. Biomater. [Internet]* **107**, 388–399, (2019). 29656599 10.1002/jbm.b.34130

[94] Liao, F., Peng, X. Y., Yang, F., Ke, Q. F., Zhu, Z. H. & Guo, Y. P. Gadolinium-doped mesoporous calcium silicate/chitosan scaffolds enhanced bone regeneration ability. *Mater. Sci. Eng. C [Internet]* **104**, 109999 (2019). 10.1016/j.msec.2019.109999 31499945

[95] Eltohamy, M., Kundu, B., Moon, J., Lee, H. Y. & Kim, H. W. Anti-bacterial zinc-doped calcium silicate cements: Bone filler. *Ceram. Int. [Internet]* **44**, 13031–13038, (2018).

[96] Çardakli, İ. S. Lanthanum oxide doped calcium silicates particles: preparation and characterization. *Süleyman Demirel Üniversitesi Fen. Bilimleri Enstitüs.ü Derg. [Internet]* **25**, 255–261, (2021).

[97] Bavya Devi, K. et al. Magnesium phosphate bioceramics for bone tissue engineering. *Chem. Rec.* **22**, e202200136 (2022). 35866502 10.1002/tcr.202200136

[98] Du, X., Lee, S. S., Blugan, G. & Ferguson, S. J. Silicon nitride as a biomedical material: an overview. *Int. J. Mol. Sci.* **23**, 6551 (2022). 35742996 10.3390/ijms23126551 PMC9224221

[99] Sainz, M. A., Serena, S., Belmonte, M., Miranzo, P. & Osendi, M. I. Protein adsorption and in vitro behavior of additively manufactured 3D-silicon nitride scaffolds intended for bone tissue engineering. *Mater. Sci. Eng. C* **115**, 110734 (2020). 10.1016/j.msec.2020.110734 32600672

[100] Du, X. et al. 3D-printed PEEK/silicon nitride scaffolds with a triply periodic minimal surface structure for spinal fusion implants. *ACS Appl. Bio Mater.* **6**, 3319–3329 (2023). 37561906 10.1021/acsabm.3c00383 PMC10445264

[101] Polley, C. et al. 3D printing of piezoelectric barium titanate-hydroxyapatite scaffolds with interconnected porosity for bone tissue engineering. *Materials* **13**, 1773 (2020). 32283869 10.3390/ma13071773 PMC7179021

[102] Tavangar, M. et al. Manufacturing and characterization of mechanical, biological and dielectric properties of hydroxyapatite-barium titanate nanocomposite scaffolds. *Ceram. Int.* **46**, 9086–9095 (2020).

[103] Tariverdian, T., Behnamghader, A., Brouki Milan, P., Barzegar-Bafrooei, H. & Mozafari, M. 3D-printed barium strontium titanate-based piezoelectric scaffolds for bone tissue engineering. *Ceram. Int.* **45**, 14029–14038 (2019).

[104] Saeidi, B., Derakhshandeh, M. R., Delshad Chermahini, M. & Doostmohammadi, A. Novel porous barium titanate/nano-bioactive glass composite with high piezoelectric coefficient for bone regeneration applications. *J. Mater. Eng. Perform.* **29**, 5420–5427 (2020).

[105] Jindal, S., Manzoor, F., Haslam, N. & Mancuso, E. 3D printed composite materials for craniofacial implants: current concepts, challenges and future directions. *Int. J. Adv. Manuf. Technol.* **112**, 635–653 (2021).

[106] Berner, A. et al. Effects of scaffold architecture on cranial bone healing. *Int. J. Oral. Maxillofac. Surg. [Internet]* **43**, 506–513, (2014). 24183512 10.1016/j.ijom.2013.05.008

[107] Zhao, Y. et al. Fabrication of gelatin methacrylate/nanohydroxyapatite microgel arrays for periodontal tissue regeneration. *Int. J. Nanomed.* **11**, 4707–4718 (2016). 10.2147/IJN.S111701 PMC5028089 27695327

[108] Cho, Y. S. et al. Assessment of osteogenesis for 3D-printed polycaprolactone/hydroxyapatite composite scaffold with enhanced exposure of hydroxyapatite using rat calvarial defect model. *Compos Sci. Technol. [Internet]* **184**, 107844 (2019).

[109] Lee, S., Choi, D., Shim, J. H. & Nam, W. Efficacy of three-dimensionally printed polycaprolactone/beta tricalcium phosphate scaffold on mandibular reconstruction. *Sci. Rep. [Internet]* **10**, 4979 (2020). 32188900 10.1038/s41598-020-61944-w PMC7080805

[110] Wu, S. C., Hsu, H. C., Hsiao, S. H. & Ho, W. F. Preparation of porous 45S5 Bioglass®-derived glass–ceramic scaffolds by using rice husk as a porogen additive. *J. Mater. Sci. Mater. Med [Internet]* **20**, 1229–1236, (2009). 19160020 10.1007/s10856-009-3690-8

[111] Chevalier, E., Chulia, D., Pouget, C. & Viana, M. Fabrication of porous substrates: a review of processes using pore forming agents in the biomaterial field. *J. Pharm. Sci. [Internet]* **97**, 1135–1154, (2008). 17688274 10.1002/jps.21059

[112] Oliveira, R. L. M. S. et al. Bioglass‐based scaffolds coated with silver nanoparticles: Synthesis, processing and antimicrobial activity. *J. Biomed. Mater. Res A [Internet]* **108**, 2447–2459, (2020). 32419306 10.1002/jbm.a.36996

[113] Fu, Q., Rahaman, M. N., Sonny Bal, B., Brown, R. F. & Day, D. E. Mechanical and in vitro performance of 13–93 bioactive glass scaffolds prepared by a polymer foam replication technique. *Acta Biomater. [Internet]* **4**, 1854–1864, (2008). 18519173 10.1016/j.actbio.2008.04.019

[114] Chen, Q. Z., Thompson, I. D. & Boccaccini, A. R. 45S5 Bioglass®-derived glass–ceramic scaffolds for bone tissue engineering. *Biomaterials [Internet]* **27**, 2414–2425, (2006). 16336997 10.1016/j.biomaterials.2005.11.025

[115] Padmanabhan, S. et al. Wollastonite/hydroxyapatite scaffolds with improved mechanical, bioactive and biodegradable properties for bone tissue engineering. *Ceram. Int. [Internet]* **39**, 619–627 (2013).

[116] de Siqueira, L. et al. Highly porous 45S5 bioglass-derived glass–ceramic scaffolds by gelcasting of foams. *J. Mater. Sci. [Internet]* **53**, 10718–10731 (2018).

[117] de Siqueira, L., de Paula, C. G., Gouveia, R. F., Motisuke, M. & de Sousa Trichês, E. Evaluation of the sintering temperature on the mechanical behavior of β-tricalcium phosphate/calcium silicate scaffolds obtained by gelcasting method. *J. Mech. Behav. Biomed. Mater. [Internet]* **90**, 635–643, (2019). 30502672 10.1016/j.jmbbm.2018.11.014

[118] Lopes, J. H. et al. Hierarchical structures of β-TCP/45S5 bioglass hybrid scaffolds prepared by gelcasting. *J. Mech. Behav. Biomed. Mater. [Internet]* **62**, 10–23, (2016). 27161958 10.1016/j.jmbbm.2016.04.028

[119] Deville, S., Saiz, E. & Tomsia, A. P. Freeze casting of hydroxyapatite scaffolds for bone tissue engineering. *Biomaterials [Internet]* **27**, 5480–5489, (2006). 16857254 10.1016/j.biomaterials.2006.06.028

[120] Paula, C. G. & de, Trichês, E. S. Preparation and characterization of β-tricalcium phosphate scaffolds by freeze casting method. *Cerâmica [Internet]* **64**, 553–558 (2018).

[121] Dash, S. R., Sarkar, R. & Bhattacharyya, S. Gel casting of hydroxyapatite with naphthalene as pore former. *Ceram. Int [Internet]* **41**, 3775–3790, (2015).

[122] González Ocampo, J. I., Escobar Sierra, D. M. & Ossa Orozco, C. P. Porous bodies of hydroxyapatite produced by a combination of the gel-casting and polymer sponge methods. *J. Adv. Res. [Internet]* **7**, 297–304, (2016). 26966570 10.1016/j.jare.2015.06.006 PMC4767808

[123] Zhang, L., Yang, G., Johnson, B. N. & Jia, X. Three-dimensional (3D) printed scaffold and material selection for bone repair. *Acta Biomater.* **84**, 16–33 (2019). 30481607 10.1016/j.actbio.2018.11.039

[124] Turnbull, G. et al. 3D bioactive composite scaffolds for bone tissue engineering. *Bioact. Mater. [Internet]* **3**, 278–314, (2018). 29744467 10.1016/j.bioactmat.2017.10.001 PMC5935790

[125] Bose, S., Vahabzadeh, S. & Bandyopadhyay, A. Bone tissue engineering using 3D printing. *Mater. Today [Internet]* **16**, 496–504, (2013).

[126] Zhang, Y. et al. Mesoporous bioactive glass nanolayer-functionalized 3D-printed scaffolds for accelerating osteogenesis and angiogenesis. *Nanoscale [Internet]* **7**, 19207–19221, (2015). 26525451 10.1039/c5nr05421d

[127] Trombetta, R., Inzana, J. A., Schwarz, E. M., Kates, S. L. & Awad, H. A. 3D printing of calcium phosphate ceramics for bone tissue engineering and drug delivery. *Ann. Biomed. Eng. [Internet]* **45**, 23–44, (2017). 27324800 10.1007/s10439-016-1678-3 PMC5173433

[128] Ngo, T. D., Kashani, A., Imbalzano, G., Nguyen, K. T. Q. & Hui, D. Additive manufacturing (3D printing): a review of materials. *methods, Appl. Chall. Compos B Eng. [Internet]* **143**, 172–196, (2018).

[129] Nik Md Noordin Kahar, N. N. F. et al. A review of bioceramics scaffolds for bone defects in different types of animal models: HA and β -TCP. *Biomed. Phys. Eng. Express* **8**, 052002 (2022). 10.1088/2057-1976/ac867f 35921834

[130] Brunello, G., Panda, S., Schiavon, L., Sivolella, S., Biasetto, L. & Del Fabbro, M. The impact of bioceramic scaffolds on bone regeneration in preclinical in vivo studies: a systematic review. *Materials* **13**, 1500 (2020). 32218290 10.3390/ma13071500 PMC7177381

[131] Tanvir, M. A. H., Khaleque, M. A., Kim, G. H., Yoo, W. Y. & Kim, Y. Y. The role of bioceramics for bone regeneration: history, mechanisms, and future perspectives. *Biomimetics* **9**, 230 (2024). 38667241 10.3390/biomimetics9040230 PMC11048714

[132] Elshazly, N., Nasr, F. E., Hamdy, A., Saied, S. & Elshazly, M. Advances in clinical applications of bioceramics in the new regenerative medicine era. *World J. Clin. Cases* **12**, 1863–1869 (2024). 38660540 10.12998/wjcc.v12.i11.1863 PMC11036528

[133] Ferraz, M. P. Bone grafts in dental medicine: an overview of autografts, allografts and synthetic materials. *Materials* **16**, 4117 (2023). 37297251 10.3390/ma16114117 PMC10254799

[134] Shao, H. et al. 3D robocasting magnesium-doped wollastonite/TCP bioceramic scaffolds with improved bone regeneration capacity in critical sized calvarial defects. *J. Mater. Chem. B [Internet]* **5**, 2941–2951, (2017). 32263987 10.1039/c7tb00217c

[135] Peng, E., Zhang, D. & Ding, J. Ceramic robocasting: recent achievements, potential, and future developments. *Adv. Mater. [Internet]*. **10**, 30 (2018). 10.1002/adma.201802404 30306642

[136] del-Mazo-Barbara, L. & Ginebra, M. P. Rheological characterisation of ceramic inks for 3D direct ink writing: a review. *J. Eur. Ceram. Soc. [Internet]* **41**, 18–33, (2021).

[137] Galván-Chacón, V. P., Eqtesadi, S., Pajares, A., Miranda, P. & Guiberteau, F. Elucidating the role of 45S5 bioglass content in the density and flexural strength of robocast β-TCP/45S5 composites. *Ceram. Int. [Internet]* **44**, 12717–12722, (2018).

[138] Roohani-Esfahani, S. I., Newman, P. & Zreiqat, H. Design and fabrication of 3D printed scaffolds with a mechanical strength comparable to cortical bone to repair large bone defects. *Sci. Rep. [Internet]* **6**, 19468, https://www.nature.com/articles/srep19468 (2016). 10.1038/srep19468 PMC4726111 26782020

[139] Marques, C. F. et al. Biphasic calcium phosphate scaffolds fabricated by direct write assembly: Mechanical, anti-microbial and osteoblastic properties. *J. Eur. Ceram. Soc. [Internet]* **37**, 359–368, (2017).

[140] Vu, A. A., Burke, D. A., Bandyopadhyay, A. & Bose, S. Effects of surface area and topography on 3D printed tricalcium phosphate scaffolds for bone grafting applications. *Addit. Manuf. [Internet]* **39**, 101870 (2021). 34307059 10.1016/j.addma.2021.101870 PMC8302005

[141] Guo, J. et al. Cold sintering: a paradigm shift for processing and integration of ceramics. *Angew. Chem. Int. Ed. [Internet]* **55**, 11457–11461 (2016). 10.1002/anie.201605443 27513705

[142] Massera, J., Fagerlund, S., Hupa, L. & Hupa, M. Crystallization mechanism of the bioactive glasses, 45S5 and S53P4. Pinckney L., editor. *J. Am. Ceramic Soc. [Internet]*. 2012, **95**, 607–613 (2012).

[143] Nommeots-Nomm, A. & Massera, J. Glass and glass-ceramic scaffolds: manufacturing methods and the impact of crystallization on in-vitro dissolution. In: *Scaffolds in Tissue Engineering - Materials, Technologies and Clinical Applications [Internet]*. InTech; 2017. http://www.intechopen.com/books/scaffolds-in-tissue-engineering-materials-technologies-and-clinical-applications/glass-and-glass-ceramic-scaffolds-manufacturing-methods-and-the-impact-of-crystallization-on-in-vitr.

[144] Ryu, H. S. et al. An improvement in sintering property of beta-tricalcium phosphate by addition of calcium pyrophosphate. *Biomaterials* **23**, 909–914 (2002). 11771710 10.1016/s0142-9612(01)00201-0

[145] Spirandeli, B. R. et al. Incorporation of 45S5 bioglass via sol-gel in β-TCP scaffolds: Bioactivity and antimicrobial activity evaluation. *Mater. Sci. Eng. C [Internet]* **131**, 112453 (2021). 10.1016/j.msec.2021.112453 34857256

[146] Sun, H. et al. 3D printing of calcium phosphate scaffolds with controlled release of antibacterial functions for jaw bone repair. *Mater. Des. [Internet]* **189**, 108540 (2020).

[147] Oliveira, R. L. M. S. et al. 3D printing of bioactive glass S53P4/sodium alginate sintering-free scaffolds. *Bioprinting [Internet]* **27**, e00226 (2022).

[148] Silva, T. L. da, Vidart, J. M. M., Silva, M. G. C. da, Gimenes, M. L. & Vieira, M. G. A. Alginate and sericin: environmental and pharmaceutical applications. In: *Biological Activities and Application of Marine Polysaccharides [Internet].* InTech; 2017. http://www.intechopen.com/books/biological-activities-and-application-of-marine-polysaccharides/alginate-and-sericin-environmental-and-pharmaceutical-applications.

[149] Kumar, A., Akkineni, A. R., Basu, B. & Gelinsky, M. Three-dimensional plotted hydroxyapatite scaffolds with predefined architecture: comparison of stabilization by alginate cross-linking versus sintering. *J. Biomater. Appl. [Internet]* **30**, 1168–1181, (2016). 26589296 10.1177/0885328215617058

[150] Pei, P., Wei, D., Zhu, M., Du, X. & Zhu, Y. The effect of calcium sulfate incorporation on physiochemical and biological properties of 3D-printed mesoporous calcium silicate cement scaffolds. *Microporous Mesoporous Mater. [Internet]* **241**, 11–20, (2017).

[151] Bertol, L. S., Schabbach, R. & Loureiro dos Santos, L. A. Different post-processing conditions for 3D bioprinted α-tricalcium phosphate scaffolds. *J. Mater. Sci. Mater. Med [Internet]* **28**, 168 (2017). 28916883 10.1007/s10856-017-5989-1

[152] Twohig, C. et al. A dual-ink 3D printing strategy to engineer pre-vascularized bone scaffolds in-vitro. *Mater. Sci. Eng. C Mater. Biol. Appl. [Internet]* **123**, 111976 (2021). 33812604 10.1016/j.msec.2021.111976 PMC12396787

[153] Hayashi, K., Yanagisawa, T., Kishida, R. & Ishikawa, K. Effects of scaffold shape on bone regeneration: tiny shape differences affect the entire system. *ACS Nano [Internet]* **16**, 11755–11768, (2022). 35833725 10.1021/acsnano.2c03776 PMC9413413

[154] Hatt, L. P., Thompson, K., Helms, J. A., Stoddart, M. J. & Armiento, A. R. Clinically relevant preclinical animal models for testing novel cranio‐maxillofacial bone 3D‐printed biomaterials. *Clin. Transl. Med. [Internet]* **12**. https://onlinelibrary.wiley.com/doi/10.1002/ctm2.690 (2022). 10.1002/ctm2.690 PMC8847734 35170248

[155] Qian, G. et al. 3D printed Zn-doped mesoporous silica-incorporated Poly-L-lactic acid scaffolds for bone repair. *Int. J. Bioprint [Internet]* **7**, 346 (2021). 33997435 10.18063/ijb.v7i2.346 PMC8114096

[156] Zhang, Y. et al. 3D gel-printed porous magnesium scaffold coated with dibasic calcium phosphate dihydrate for bone repair in vivo. *J. Orthop. Transl. [Internet]* **33**, 13–23, (2022). 10.1016/j.jot.2021.11.005 PMC8819133 35198379

[157] Ballouze, R. et al. Biocompatible magnesium-doped biphasic calcium phosphate for bone regeneration. *J. Biomed. Mater. Res. B Appl. Biomater. [Internet]* **109**, 1426–1435 (2021). 33484103 10.1002/jbm.b.34802

[158] Li, S. et al. Evaluation of highly carbonated hydroxyapatite bioceramic implant coatings with hierarchical micro-/nanorod topography optimized for osseointegration. *Int. J. Nanomed. [Internet]* **ume 13**, 3643–3659 (2018). 10.2147/IJN.S159989 PMC6027846 29983560

[159] Zhuang, Y. et al. A biomimetic zinc alloy scaffold coated with brushite for enhanced cranial bone regeneration. *ACS Biomater. Sci. Eng. [Internet]* **7**, 893–903 (2021). 33715369 10.1021/acsbiomaterials.9b01895

[160] Wang, B. et al. The study of angiogenesis stimulated by multivalent peptide ligand-modified alginate. *Colloids Surf. B Biointerfaces [Internet]* **154**, 383–390 (2017). 28384617 10.1016/j.colsurfb.2017.03.049

[161] Hao, D. et al. Rapid endothelialization of small diameter vascular grafts by a bioactive integrin-binding ligand specifically targeting endothelial progenitor cells and endothelial cells. *Acta Biomater. [Internet]* **108**, 178–193, (2020). 32151698 10.1016/j.actbio.2020.03.005 PMC8012081

[162] Xu, Z. et al. Poly(Dopamine) coating on 3D-printed Poly-Lactic-Co-Glycolic Acid/β-tricalcium phosphate scaffolds for bone tissue engineering. *Molecules [Internet]* **24**, 4397 (2019). 31810169 10.3390/molecules24234397 PMC6930468

[163] Ho, C. C. et al. Effect of mussel-inspired polydopamine on the reinforced properties of 3D printed β-tricalcium phosphate/polycaprolactone scaffolds for bone regeneration. *J. Mater. Chem. B [Internet]* **11**, 72–82 (2023). 10.1039/d2tb01995g 36373587

[164] Nayak, V. V. et al. Three-dimensional printing bioceramic scaffolds using direct-ink-writing for craniomaxillofacial bone regeneration. *Tissue Eng. C Methods [Internet]* **29**, 332–345 (2023). 10.1089/ten.tec.2023.0082 PMC10495199 37463403

[165] Zhang, F. et al. A review of 3D printed porous ceramics. *J. Eur. Ceram. Soc. [Internet]* **42**, 3351–3373 (2022).

[166] Swanson, W. B. et al. Macropore design of tissue engineering scaffolds regulates mesenchymal stem cell differentiation fate. *Biomaterials* **272**, 120769 (2021). 33798961 10.1016/j.biomaterials.2021.120769 PMC8068670

[167] Yang, Z. et al. Biomechanical effects of 3D-printed bioceramic scaffolds with porous gradient structures on the regeneration of alveolar bone defect: a comprehensive study. *Front Bioeng. Biotechnol.* **10**, 882631 (2022). 35694236 10.3389/fbioe.2022.882631 PMC9177945

[168] Entezari, A. et al. Architectural design of 3D printed scaffolds controls the volume and functionality of newly formed bone. *Adv. Healthc. Mater [Internet]*. **8**. https://onlinelibrary.wiley.com/doi/10.1002/adhm.201801353 (2019). 10.1002/adhm.201801353 30536610

[169] Eichholz, K. F. et al. Scaffold microarchitecture regulates angiogenesis and the regeneration of large bone defects. *Biofabrication [Internet]* **14**, 045013 (2022). 10.1088/1758-5090/ac88a1 35947963

[170] Hollister, S. J. Porous scaffold design for tissue engineering. *Nat. Mater.* **4**, 518–524 (2005). 16003400 10.1038/nmat1421

[171] Maliha, S. G. et al. Bone tissue engineering in the growing calvaria using dipyridamole-coated, three-dimensionally–printed bioceramic scaffolds: construct optimization and effects on cranial suture patency. *Plast. Reconstr. Surg. [Internet]* **145**, 337e–347ee, (2020). 31985634 10.1097/PRS.0000000000006483 PMC7212767

[172] Ellermann, E., Meyer, N., Cameron, R. E. & Best S. M. In vitro angiogenesis in response to biomaterial properties for bone tissue engineering: a review of the state of the art. *Regen Biomater [Internet]*. **10**. https://academic.oup.com/rb/article/doi/10.1093/rb/rbad027/7087108 (2023). 10.1093/rb/rbad027 PMC10112962 37081860

[173] Marques, A., Miranda, G., Silva, F., Pinto, P. & Carvalho, Ó. Review on current limits and potentialities of technologies for biomedical ceramic scaffolds production. *J. Biomed. Mater. Res B Appl. Biomater. [Internet]* **109**, 377–393, (2021). 32924277 10.1002/jbm.b.34706

[174] Ge, R., Xun, C., Yang, J., Jia, W. & Li, Y. In vivo therapeutic effect of wollastonite and hydroxyapatite on bone defect. *Biomed. Mater. [Internet]* **14**, 065013 (2019). 31491772 10.1088/1748-605X/ab4238

[175] Barba, A. et al. Osteogenesis by foamed and 3D-printed nanostructured calcium phosphate scaffolds: effect of pore architecture. *Acta Biomater. [Internet]* **79**, 135–147, (2018). 30195084 10.1016/j.actbio.2018.09.003

[176] Fu, Z., Ouyang, L., Xu, R., Yang, Y. & Sun, W. Responsive biomaterials for 3D bioprinting: a review. *Mater. Today [Internet]* **52**, 112–132, (2022).

[177] Gu, Y. et al. Three-dimensional printed Mg-Doped β-TCP bone tissue engineering scaffolds: effects of magnesium ion concentration on osteogenesis and angiogenesis in vitro. *Tissue Eng. Regen. Med [Internet]* **16**, 415–429 (2019). 31413945 10.1007/s13770-019-00192-0 PMC6675836

[178] Kim, S. E., Shim, K. M., Jang, K., Shim, J. H. & Kang, S. S. Three-dimensional printing-based reconstruction of a maxillary bone defect in a dog following tumor removal. *Vivo (Brooklyn) [Internet]* **32**, 63–70, (2018). 10.21873/invivo.11205 PMC5892642 29275300

[179] Liu, R. et al. Effects of pore size on the mechanical and biological properties of stereolithographic 3D printed HAp bioceramic scaffold. *Ceram. Int. [Internet]* **47**, 28924–28931 (2021).

[180] Mirkhalaf, M. et al. Redefining architectural effects in 3D printed scaffolds through rational design for optimal bone tissue regeneration. *Appl. Mater. Today [Internet]* **25**, 101168 (2021).

[181] Ghayor, C., Weber, F. E. Osteoconductive microarchitecture of bone substitutes for bone regeneration revisited. *Front. Physiol. [Internet]*. **9**. https://www.frontiersin.org/article/10.3389/fphys.2018.00960/full (2018). 10.3389/fphys.2018.00960 PMC6060436 30072920

[182] Qin, H. et al. 3D printed bioceramic scaffolds: adjusting pore dimension is beneficial for mandibular bone defects repair. *J. Tissue Eng. Regen. Med [Internet]* **16**, 409–421, (2022). 35156316 10.1002/term.3287

[183] Lee, S. J. et al. Development of a three-dimensionally printed scaffold grafted with bone forming peptide-1 for enhanced bone regeneration with in vitro and in vivo evaluations. *J. Colloid Interface Sci. [Internet]* **539**, 468–480, (2019). 30611042 10.1016/j.jcis.2018.12.097

[184] Lee, D. J. et al. Effect of pore size in bone regeneration using polydopamine‐laced hydroxyapatite collagen calcium silicate scaffolds fabricated by 3D mould printing technology. *Orthod. Craniofac Res. [Internet]* **22**, 127–133, (2019). 31074145 10.1111/ocr.12261 PMC6512819

[185] Diao, J. et al. 3D‐plotted beta‐tricalcium phosphate scaffolds with smaller pore sizes improve in vivo bone regeneration and biomechanical properties in a critical‐sized calvarial defect rat model. *Adv Healthc Mater [Internet]*. **7**. https://onlinelibrary.wiley.com/doi/10.1002/adhm.201800441 (2018). 10.1002/adhm.201800441 PMC6355155 30044555

[186] Wu, F. et al. Integrating pore architectures to evaluate vascularization efficacy in silicate-based bioceramic scaffolds. *Regen. Biomater.* **9**, rbab077 (2022). 35480859 10.1093/rb/rbab077 PMC9039507

[187] Barba, A. et al. Osteoinduction by foamed and 3D-printed calcium phosphate scaffolds: effect of nanostructure and pore architecture. *ACS Appl. Mater. Interfaces [Internet]* **9**, 41722–41736, (2017). 29116737 10.1021/acsami.7b14175

[188] Bidan, C. M. et al. Geometry as a factor for tissue growth: towards shape optimization of tissue engineering scaffolds. *Adv. Health. Mater. [Internet]* **2**, 186–194 (2013). 10.1002/adhm.201200159 23184876

[189] Subbiah, R. et al. 3D printing of microgel‐loaded modular microcages as instructive scaffolds for tissue engineering. *Adv. Mater. [Internet]*. **32**. (2020). 10.1002/adma.202001736 32700332

[190] Li, T. et al. 3D printing of hot dog‐like biomaterials with hierarchical architecture and distinct bioactivity. *Adv. Sci. [Internet]*. 2019 Oct 8;6. https://onlinelibrary.wiley.com/doi/10.1002/advs.201901146 (2019). 10.1002/advs.201901146 PMC6774059 31592134

[191] Korn, P. et al. 3D printing of bone grafts for cleft alveolar osteoplasty – in vivo evaluation in a preclinical model. *Front Bioeng. Biotechnol. [Internet]*. 2020 Mar 25;8. https://www.frontiersin.org/article/10.3389/fbioe.2020.00217/full (2020). 10.3389/fbioe.2020.00217 PMC7109264 32269989

[192] Kilian, D. et al. 3D printing of patient-specific implants for osteochondral defects: workflow for an MRI-guided zonal design. *Biodes Manuf. [Internet]* **4**, 818–832, (2021).

[193] Sharma, N. et al. Quantitative assessment of point-of-care 3D-printed patient-specific polyetheretherketone (PEEK) cranial implants. *Int. J. Mol. Sci. [Internet]*. 2021 Aug 7;22. http://www.ncbi.nlm.nih.gov/pubmed/34445228 (2021). 10.3390/ijms22168521 PMC8395180 34445228

[194] Gelețu, G. et al. Customized 3D-printed titanium mesh developed for an aesthetic zone to regenerate a complex bone defect resulting after a deficient odontectomy: a case report. *Medicina (B Aires) [Internet]* **58**, 1192 (2022). 10.3390/medicina58091192 PMC9504411 36143869

[195] Gubin, A. V. et al. Challenges and perspectives in the use of additive technologies for making customized implants for traumatology and orthopedics. *Biomed. Eng. (NY)* **50**, 285–289 (2016).

[196] Charbonnier, B., Hadida, M. & Marchat, D. Additive manufacturing pertaining to bone: hopes, reality and future challenges for clinical applications. *Acta Biomater.* **121**, 1–28 (2021). 33271354 10.1016/j.actbio.2020.11.039

[197] Ivanovski, S. et al. 3D printing for bone regeneration: challenges and opportunities for achieving predictability. *Periodontol 2000* **93**, 358–384 (2023). 37823472 10.1111/prd.12525

[198] Center for Devices and Radiological Health. Technical Considerations for Additive Manufactured Medical Devices, U.S. Food and Drug Administration. [Internet]. 2020 [cited 2024 Jul 21]. https://www.fda.gov/regulatory-information/search-fda-guidance-documents/technical-considerations-additive-manufactured-medical-devices.

[199] Lee, S. et al. Emerging technology as a key enabler for modernizing pharmaceutical manufacturing. *PDA J. Pharm. Sci. Technol.* **71**, 66–67 (2017). 28396563 10.5731/pdajpst.2017.001100

[200] BG, P. K., Mehrotra, S., Marques, S. M., Kumar, L. & Verma, R. 3D printing in personalized medicines: a focus on applications of the technology. *Mater. Today Commun.* **35**, 105875 (2023).

[201] Al-Litani, K., Ali, T., Robles Martinez, P. & Buanz, A. 3D printed implantable drug delivery devices for women’s health: Formulation challenges and regulatory perspective. *Adv. Drug Deliv. Rev.* **198**, 114859 (2023). 37149039 10.1016/j.addr.2023.114859

[202] Shah, S. R., et al. A composite critical-size rabbit mandibular defect for evaluation of craniofacial tissue regeneration. *Nat. Protoc. [Internet]* **11**, 1989–2009 (2016). 10.1038/nprot.2016.122 27658014

[203] Ma, H., Feng, C., Chang, J. & Wu, C. 3D-printed bioceramic scaffolds: from bone tissue engineering to tumor therapy. *Acta Biomater. [Internet]* **79**, 37–59, (2018). 30165201 10.1016/j.actbio.2018.08.026

[204] Bruyas, A. et al. Systematic characterization of 3D-printed PCL/β-TCP scaffolds for biomedical devices and bone tissue engineering: Influence of composition and porosity. *J. Mater. Res [Internet]* **33**, 1948–1959 (2018). 30364693 10.1557/jmr.2018.112 PMC6197810

[205] Francisco, I. et al. Three-dimensional impression of biomaterials for alveolar graft: scoping review. *J. Funct. Biomater. [Internet]* **14**, 76 (2023). 36826875 10.3390/jfb14020076 PMC9961517

[206] Tang, Z., Li, X., Tan, Y., Fan, H. & Zhang, X. The material and biological characteristics of osteoinductive calcium phosphate ceramics. *Regen. Biomater. [Internet]* **5**, 43–59, (2018). 29423267 10.1093/rb/rbx024 PMC5798025

[207] Dang, W. et al. A bifunctional scaffold with CuFeSe2 nanocrystals for tumor therapy and bone reconstruction. *Biomaterials [Internet]* **160**, 92–106, (2018). 29407343 10.1016/j.biomaterials.2017.11.020

[208] Zhang, W. et al. 3D-printed scaffolds with synergistic effect of hollow-pipe structure and bioactive ions for vascularized bone regeneration. *Biomaterials [Internet]* **135**, 85–95 (2017). 28499127 10.1016/j.biomaterials.2017.05.005

[209] Ye, J. et al. The interaction between intracellular energy metabolism and signaling pathways during osteogenesis. *Front. Mol. Biosci.* **8**, 807487 (2022). 35155568 10.3389/fmolb.2021.807487 PMC8832142

[210] Yuan, X. et al. Recent advances in 3D printing of smart scaffolds for bone tissue engineering and regeneration. *Adv. Mater.* **36**, 2403641 (2024). 10.1002/adma.202403641 38861754

[211] Yang, J., Ueharu, H. & Mishina, Y. Energy metabolism: a newly emerging target of BMP signaling in bone homeostasis. *Bone* **138**, 115467 (2020). 32512164 10.1016/j.bone.2020.115467 PMC7423769

[212] Kang, Z. et al. Metabolic regulation by biomaterials in osteoblast. *Front. Bioeng. Biotechnol.* **11**, 1184463 (2023). 37324445 10.3389/fbioe.2023.1184463 PMC10265685

[213] Rosenberg, N. The theoretical context of biophysical stimulation of osteoblasts. In: *Biophysical Osteoblast Stimulation for Bone Grafting and Regeneration* 3–12 (Springer International Publishing, 2023).

[214] Na, J. et al. Extracellular matrix stiffness as an energy metabolism regulator drives osteogenic differentiation in mesenchymal stem cells. *Bioact. Mater.* **35**, 549–563 (2024). 38434800 10.1016/j.bioactmat.2024.02.003 PMC10909577

[215] Rahimnejad, M., Rezvaninejad, R., Rezvaninejad, R. & França, R. Biomaterials in bone and mineralized tissue engineering using 3D printing and bioprinting technologies. *Biomed. Phys. Eng. Express* **7**, 062001 (2021). 10.1088/2057-1976/ac21ab 34438382

[216] Michigami, T., Kawai, M., Yamazaki, M. & Ozono, K. Phosphate as a signaling molecule and its sensing mechanism. *Physiol. Rev.* **98**, 2317–2348 (2018). 30109818 10.1152/physrev.00022.2017

[217] Islam, M. S. Calcium signaling: from basic to bedside. *Adv. Exp. Med. Biol.* **1131**, 1–6 (2020). 31646504 10.1007/978-3-030-12457-1_1

[218] Suzuki, O., Shiwaku, Y. & Hamai, R. Octacalcium phosphate bone substitute materials: Comparison between properties of biomaterials and other calcium phosphate materials. *Dent. Mater. J.* **39**, 187–199 (2020). 32161239 10.4012/dmj.2020-001

[219] Danoux, C. B. S. S. et al. Elucidating the individual effects of calcium and phosphate ions on hMSCs by using composite materials. *Acta Biomater.* **17**, 1–15 (2015). 25676583 10.1016/j.actbio.2015.02.003

[220] Barradas, A. M. C. et al. A calcium-induced signaling cascade leading to osteogenic differentiation of human bone marrow-derived mesenchymal stromal cells. *Biomaterials* **33**, 3205–3215 (2012). 22285104 10.1016/j.biomaterials.2012.01.020

[221] Tada, H., Nemoto, E., Foster, B. L., Somerman, M. J. & Shimauchi, H. Phosphate increases bone morphogenetic protein-2 expression through cAMP-dependent protein kinase and ERK1/2 pathways in human dental pulp cells. *Bone* **48**, 1409–1416 (2011). 21419244 10.1016/j.bone.2011.03.675

[222] Wang, X. et al. Calcium phosphate-based materials regulate osteoclast-mediated osseointegration. *Bioact. Mater.* **6**, 4517–4530 (2021). 34632163 10.1016/j.bioactmat.2021.05.003 PMC8484898

[223] Si, J. et al. Osteopontin in bone metabolism and bone diseases. *Med. Sci. Monit.* **26**, e919159 (2020). 31996665 10.12659/MSM.919159 PMC7003659

[224] Vermeulen, S. et al. An in vitro model system based on calcium- and phosphate ion-induced hMSC spheroid mineralization. *Mater. Today Bio* **23**, 100844 (2023). 38033367 10.1016/j.mtbio.2023.100844 PMC10682137

[225] Xiao, D. et al. The role of calcium phosphate surface structure in osteogenesis and the mechanisms involved. *Acta Biomater.* **106**, 22–33 (2020). 31926336 10.1016/j.actbio.2019.12.034

[226] Guo, X. et al. The implication of the notch signaling pathway in biphasic calcium phosphate ceramic‐induced ectopic bone formation: a preliminary experiment. *J. Biomed. Mater. Res A* **108**, 1035–1044 (2020). 31925903 10.1002/jbm.a.36878

[227] Sugiatno, E., Herminajeng, E. & Sosroseno, W. The role of prostaglandin E2 on osteoblast proliferation induced by hydroxyapatite. *J. Biosci. Med (Irvine)* **08**, 42–55 (2020).

[228] Miroshnichenko, L. A., Polyakova, T. Y. U., Litvinova, L. S. & Khlusov, I. A. Review of local cellular and molecular processes of bone tissue regeneration induced by calcium phosphate materials. *Cell Tissue Biol.* **18**, 148–162 (2024).

[229] Zhu, M., Zhang, R., Mao, Z., Fang, J. & Ren, F. Topographical biointerface regulating cellular functions for bone tissue engineering. *Biosurf. Biotribol.* **8**, 165–187 (2022).

[230] Kermani, F., Kargozar, S., Dorozhkin, S. V., Mollazadeh, S. Calcium phosphate bioceramics for improved angiogenesis. In: *Biomaterials for Vasculogenesis and Angiogenesis* 185–203 (Elsevier, 2022).

[231] Kumar, A. et al. Synergistic effect of biphasic calcium phosphate and platelet-rich fibrin attenuate markers for inflammation and osteoclast differentiation by suppressing NF-κB/MAPK signaling pathway in chronic periodontitis. *Molecules* **26**, 6578 (2021). 34770985 10.3390/molecules26216578 PMC8587053

[232] Spagnuolo, G. et al. An in-vitro study investigating the effect of air-abrasion bioactive glasses on dental adhesion, cytotoxicity and odontogenic gene expression. *Dent. Mater.* **37**, 1734–1750 (2021). 34561100 10.1016/j.dental.2021.09.004

[233] Hohenbild, F. et al. An in vitro evaluation of the biological and osteogenic properties of magnesium-doped bioactive glasses for application in bone tissue engineering. *Int J. Mol. Sci.* **22**, 12703 (2021). 34884519 10.3390/ijms222312703 PMC8657676

[234] Turner, J. et al. The effect of Si species released from bioactive glasses on cell behaviour: a quantitative review. *Acta Biomater.* **170**, 39–52 (2023). 37714247 10.1016/j.actbio.2023.09.012

[235] Huang, D. et al. Strontium-substituted sub-micron bioactive glasses inhibit ostoclastogenesis through suppression of RANKL-induced signaling pathway. *Regen. Biomater.* **7**, 303–311 (2020). 32523732 10.1093/rb/rbaa004 PMC7266663

[236] Zhang, C., Yuan, Y., Fang, L. & Xuan, Y. Promotion of osteogenesis by bioactive glass–ceramic coating: Possible involvement of the Hedgehog signaling pathway. *J. Orthop. Sci.* **24**, 731–736 (2019). 30638689 10.1016/j.jos.2018.12.006

[237] Williams, D. F. Biocompatibility pathways and mechanisms for bioactive materials: the bioactivity zone. *Bioact. Mater.* **10**, 306–322 (2022). 34901548 10.1016/j.bioactmat.2021.08.014 PMC8636667

[238] Bogoyevitch, M. A., Ngoei, K. R. W., Zhao, T. T., Yeap, Y. Y. C. & Ng, D. C. H. c-Jun N-terminal kinase (JNK) signaling: recent advances and challenges. *Biochim. Biophys. Acta (BBA) - Proteins Proteom.* **1804**, 463–475 (2010). 10.1016/j.bbapap.2009.11.002 19900593

[239] Gong, W., Dong, Y., Wang, S., Gao, X. & Chen, X. A novel nano-sized bioactive glass stimulates osteogenesis via the MAPK pathway. *RSC Adv.* **7**, 13760–13767 (2017).

[240] Li, J. et al. Ion release behavior of vanadium-doped mesoporous bioactive glass particles and the effect of the released ions on osteogenic differentiation of BMSCs *via* the FAK/MAPK signaling pathway. *J. Mater. Chem. B* **9**, 7848–7865 (2021). 34586154 10.1039/d1tb01479j

[241] Fellenberg, J. et al. Bioactive glass selectively promotes cytotoxicity towards giant cell tumor of bone derived neoplastic stromal cells and induces MAPK signalling dependent autophagy. *Bioact. Mater.* **15**, 456–468 (2022). 35386334 10.1016/j.bioactmat.2022.02.021 PMC8958388

[242] Łukowicz, K. et al. The role of CaO/SiO2 ratio and P2O5 content in gel-derived bioactive glass-polymer composites in the modulation of their bioactivity and osteoinductivity in human BMSCs. *Mater. Sci. Eng. C* **109**, 110535 (2020). 10.1016/j.msec.2019.110535 32228933

[243] Zheng, K., Niu, W., Lei, B. & Boccaccini, A. R. Immunomodulatory bioactive glasses for tissue regeneration. *Acta Biomater.* **133**, 168–186 (2021). 34418539 10.1016/j.actbio.2021.08.023

[244] Khotib, J., Gani, M. A. & Budiatin, A. S. Lestari MLAD, Rahadiansyah E, Ardianto C. Signaling pathway and transcriptional regulation in osteoblasts during bone healing: direct involvement of hydroxyapatite as a biomaterial. *Pharmaceuticals* **14**, 615 (2021). 34206843 10.3390/ph14070615 PMC8308723

[245] Liang, H. et al. Gold nanoparticles-loaded hydroxyapatite composites guide osteogenic differentiation of human mesenchymal stem cells through Wnt/β-catenin signaling pathway. *Int. J. Nanomed.* **14**, 6151–6163 (2019). 10.2147/IJN.S213889 PMC6683960 31447557

[246] Kuntin, D., Gosling, N., Wood, D. & Genever, P. Wnt signalling in mesenchymal stem cells is heightened in response to plasma sprayed hydroxyapatite coatings. *Osteoarthr. Cartil.* **26**, S146 (2018).

[247] Wang, J. et al. Nano-hydroxyapatite coating promotes porous calcium phosphate ceramic-induced osteogenesis via BMP/Smad signaling pathway. *Int. J. Nanomed.* **14**, 7987–8000 (2019). 10.2147/IJN.S216182 PMC6781424 31632013

[248] Jean Gabriel Garcia-Diaz. *WNT ligand-specific signaling in bone* (John Hopkins University, 2023).

[249] Baron, R. & Kneissel, M. WNT signaling in bone homeostasis and disease: from human mutations to treatments. *Nat. Med* **19**, 179–192 (2013). 23389618 10.1038/nm.3074

[250] Huang, K. et al. Wnt10b regulates osteogenesis of adipose-derived stem cells through Wnt/β-catenin signalling pathway in osteoporosis. *Cell Prolif.* **57**, e13522 (2024). 37340715 10.1111/cpr.13522 PMC10771102

[251] Zhou, J., Zhao, L., Li, B. & Han, Y. Nanorod diameter modulated osteogenic activity of hierarchical micropore/nanorod-patterned coatings via a Wnt/β-catenin pathway. *Nanomedicine* **14**, 1719–1731 (2018). 29665441 10.1016/j.nano.2018.04.006

[252] Ha, S. W., Park, J., Habib, M. M. & Beck, G. R. Nano-hydroxyapatite stimulation of gene expression requires Fgf receptor, phosphate transporter, and Erk1/2 signaling. *ACS Appl. Mater. Interfaces* **9**, 39185–39196 (2017). 29045789 10.1021/acsami.7b12029 PMC10336561

[253] Song, Y. et al. Zinc silicate/nano-hydroxyapatite/collagen scaffolds promote angiogenesis and bone regeneration via the p38 MAPK pathway in activated monocytes. *ACS Appl. Mater. Interfaces* **12**, 16058–16075 (2020). 32182418 10.1021/acsami.0c00470

[254] Xu, D. et al. Tailorable hierarchical structures of biomimetic hydroxyapatite micro/nano particles promoting endocytosis and osteogenic differentiation of stem cells. *Biomater. Sci.* **8**, 3286–3300 (2020). 32490486 10.1039/d0bm00443j

[255] Hiragami, F., Akiyama, J., Koike, Y. & Kano, Y. Enhancement of hydroxyapatite‐mediated three‐dimensional‐like proliferation of mouse fibroblasts by heat treatment: Effects of heat shock‐induced p38 MAPK pathway. *J. Biomed. Mater. Res. A* **74A**, 705–711 (2005). 10.1002/jbm.a.30362 16035075

[256] Yang, C. et al. Stimulation of osteogenesis and angiogenesis by micro/nano hierarchical hydroxyapatite *via* macrophage immunomodulation. *Nanoscale* **11**, 17699–17708 (2019). 31545331 10.1039/c9nr05730g

[257] Zhuang, Y. et al. Promoting vascularized bone regeneration via strontium-incorporated hydroxyapatite bioceramic. *Mater. Des.* **234**, 112313 (2023).

[258] Mestres, G. et al. Inflammatory response to nano- and microstructured hydroxyapatite. *PLoS One* **10**, e0120381 (2015). 25837264 10.1371/journal.pone.0120381 PMC4383585

[259] Sparks, D. S. et al. A preclinical large-animal model for the assessment of critical-size load-bearing bone defect reconstruction. *Nat. Protoc. [Internet]* **15**, 877–924, (2020). 32060491 10.1038/s41596-019-0271-2

[260] Lee, J. S. et al. Osteogenesis of 3D-Printed PCL/TCP/bdECM scaffold using adipose-derived stem cells aggregates; an experimental study in the canine mandible. *Int. J. Mol. Sci. [Internet]*. **22**. http://www.ncbi.nlm.nih.gov/pubmed/34063742 (2021). 10.3390/ijms22115409 PMC8196585 34063742

[261] Kotagudda Ranganath, S., Schlund, M., Delattre, J., Ferri, J. & Chai, F. Bilateral double site (calvarial and mandibular) critical-size bone defect model in rabbits for evaluation of a craniofacial tissue engineering constructs. *Mater. Today Bio* **14**, 100267 (2022). 35514436 10.1016/j.mtbio.2022.100267 PMC9061786

[262] Lopez, C. D. et al. Regeneration of a pediatric alveolar cleft model using three-dimensionally printed bioceramic scaffolds and osteogenic agents: comparison of dipyridamole and rhBMP-2. *Plast. Reconstr. Surg.* **144**, 358–370 (2019). 31348344 10.1097/PRS.0000000000005840 PMC6668366

[263] Carrel, J., Wiskott, A., Scherrer, S. & Durual, S. Large bone vertical augmentation using a three‐dimensional printed TCP/HA bone graft: a pilot study in dog mandible. *Clin. Implant Dent. Relat. Res. [Internet]* **18**, 1183–1192, (2016). 26899497 10.1111/cid.12394

[264] Shen, C. et al. Three-dimensional printing for craniofacial bone tissue engineering. *Tissue Eng. A [Internet]* **26**, 1303–1311, https://www.liebertpub.com/doi/10.1089/ten.tea.2020.0186 (2020). 10.1089/ten.tea.2020.0186 PMC7759279 32842918

[265] Raymond, Y. et al. 3D printing with star-shaped strands: a new approach to enhance in vivo bone regeneration. *Biomater. Adv. [Internet]* **137**, 212807 (2022). 35929234 10.1016/j.bioadv.2022.212807

[266] Zhang, J. et al. Biodegradable metals for bone defect repair: a systematic review and meta-analysis based on animal studies. *Bioact. Mater.* **6**, 4027–4052 (2021). 33997491 10.1016/j.bioactmat.2021.03.035 PMC8089787

[267] Simunovic, F. & Finkenzeller, G. Vascularization strategies in bone tissue engineering. *Cells* **10**, 1749 (2021). 34359919 10.3390/cells10071749 PMC8306064

[268] Rahimnejad, M. et al. Engineered biomimetic membranes for organ-on-a-chip. *ACS Biomater. Sci. Eng.* **8**, 5038–5059 (2022). 36347501 10.1021/acsbiomaterials.2c00531

[269] Shiwarski, D. J., Hudson, A. R., Tashman, J. W. & Feinberg, A. W. Emergence of FRESH 3D printing as a platform for advanced tissue biofabrication. *APL Bioeng.* **5**, 010904 (2021). 33644626 10.1063/5.0032777 PMC7889293

[270] Madadian, E. et al. In-foam bioprinting: an embedded bioprinting technique with self-removable support bath. *Small Sci.* **4**, 2300280 (2024). 10.1002/smsc.202300280 PMC11935082 40213570

[271] Moeun, B. et al. Vascularizing a human-scale bioartificial pancreas using sacrificial embedded 3D printing into self-healing alginate. *Transplantation* **107**, 60–60 (2023).

[272] Kolomenskaya, E., Butova, V., Poltavskiy, A., Soldatov, A. & Butakova, M. Application of artificial intelligence at all stages of bone tissue engineering. *Biomedicines* **12**, 76 (2023). 38255183 10.3390/biomedicines12010076 PMC10813365

[273] Rahimnejad, M. et al. Stimuli-responsive biomaterials: smart avenue toward 4D bioprinting. *Crit. Rev. Biotechnol.* **44**, 860–891 (2024). 37442771 10.1080/07388551.2023.2213398

[274] Golafshan, N. et al. Tough magnesium phosphate-based 3D-printed implants induce bone regeneration in an equine defect model. *Biomaterials [Internet]* **261**, 120302 (2020). 32932172 10.1016/j.biomaterials.2020.120302 PMC7116184

[275] Zhang, W. et al. 3D printed composite scaffolds with dual small molecule delivery for mandibular bone regeneration. *Biofabrication [Internet]* **12**, 035020 (2020). 32369796 10.1088/1758-5090/ab906e PMC8059098

[276] Martínez-Vázquez, F. J., Cabañas, M. V., Paris, J. L., Lozano, D. & Vallet-Regí, M. Fabrication of novel Si-doped hydroxyapatite/gelatine scaffolds by rapid prototyping for drug delivery and bone regeneration. *Acta Biomater. [Internet]* **15**, 200–209 (2015). 25560614 10.1016/j.actbio.2014.12.021

[277] Arbex, L. et al. Physio-mechanical and biological effects due to surface area modifications of 3D printed β-tri- calcium phosphate: an in vitro study. Annals of 3D printed. *Medicine* **8**, 100078 (2022).

[278] Wang, J. et al. Fabrication and biological evaluation of 3D-printed calcium phosphate ceramic scaffolds with distinct macroporous geometries through digital light processing technology. *Regen. Biomater.* **9**, rbac005 (2022). 35668922 10.1093/rb/rbac005 PMC9160879

