# Supplementary figures and images for: Personalized bioceramic grafts for craniomaxillofacial bone regeneration
Source: Int J Oral Sci. 2024 Oct 31;16:62. doi: 10.1038/s41368-024-00327-7 (PMC11528123; doi:10.1038/s41368-024-00327-7)

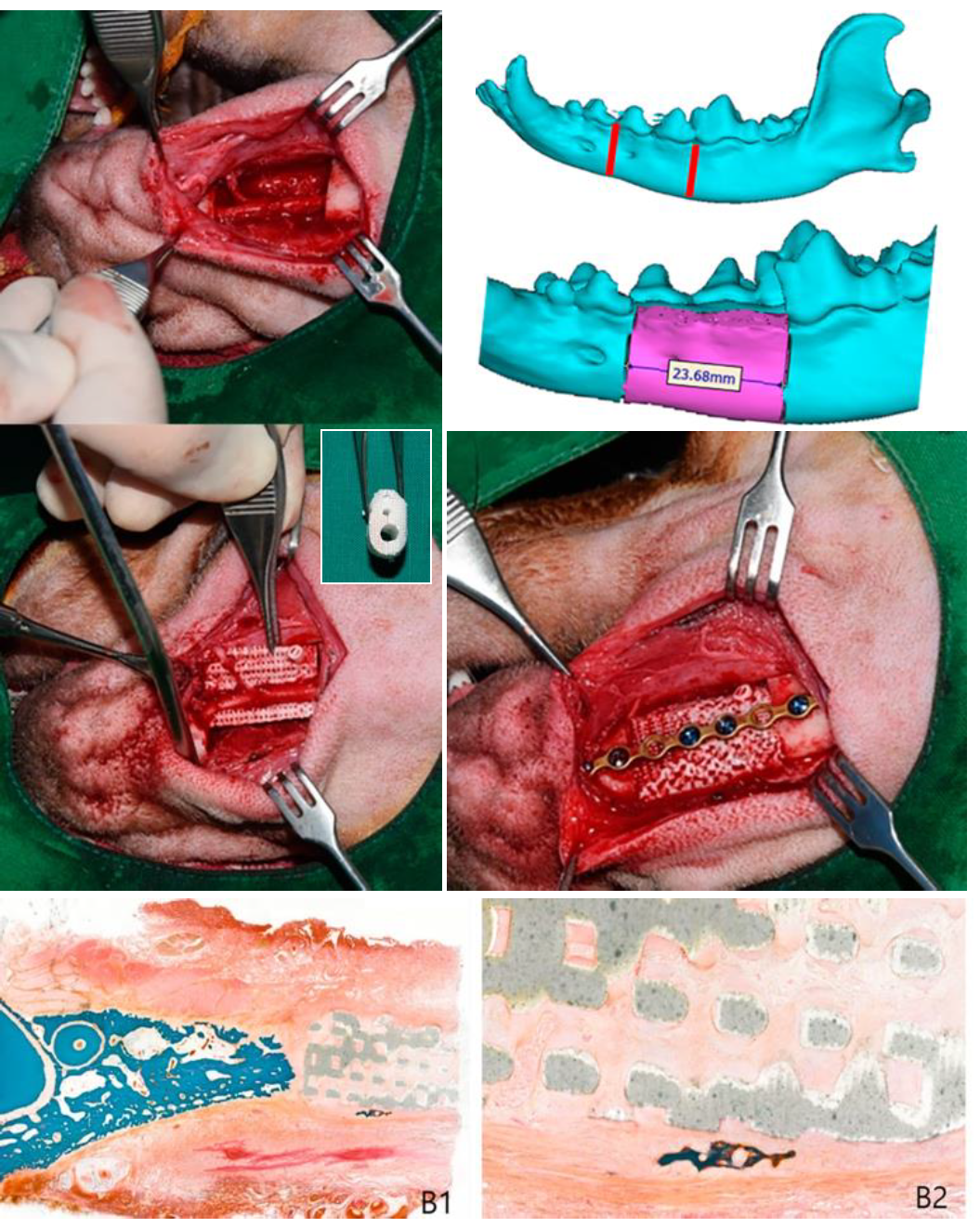

Supplement: Supplementary file 1 — Figure1S. Supplemental Figure [file 41368_2024_327_MOESM1_ESM.tif]
