# Supplementary material for: Personalized bioceramic grafts for craniomaxillofacial bone regeneration
Source: Int J Oral Sci. 2024 Oct 31;16:62. doi: 10.1038/s41368-024-00327-7 (PMC11528123; doi:10.1038/s41368-024-00327-7)
Supplement: Supplementary file 2 — Supplemental Information [file 41368_2024_327_MOESM2_ESM.docx]

**Supplementary Information**

**Figure 1S.** *In vivo* assessment for 3D printed bioceramic scaffolds. Surgical procedures in beagle dogs for placement of a personalized scaffold followed by histological examination. Figure adapted from Lee *et al*.^261^. Open access article distributed under the terms and conditions of the Creative Commons Attribution (CC BY) license (<https://creativecommons.org/licenses/by/4.0/>).
